# Phenylpropanoid amides from *Solanum rostratum* and their phytotoxic activities against *Arabidopsis thaliana*

**DOI:** 10.3389/fpls.2023.1174844

**Published:** 2023-04-12

**Authors:** Zhixiang Liu, Xiaoqing Ma, Nan Zhang, Linlin Yuan, Hongrui Yin, Lingling Zhang, Tong An, Yubin Xu

**Affiliations:** ^1^ College of Bioscience and Biotechnology, Shenyang Agricultural University, Shenyang, China; ^2^ Department of Pharmacy, Taizhou Central Hospital (Taizhou University Hospital), Taizhou, China

**Keywords:** invasive plant, isolate, phenylpropanoid amides, phytotoxic activities, *Solanum rostratum*, *Arabidopsis thaliana*

## Abstract

**Introduction:**

*Solanum rostratum*, an annual malignant weed, has seriously damaged the ecological environment and biodiversity of invasion area. This alien plant gains a competitive advantage by producing some new phytotoxic substances to inhibit the growth of native plants, thus achieving successful invasion. However, the chemical structures, inhibitory functions and action mechanisms of phytotoxic substances of *S. rostratum* remain unclear.

**Methods:**

In this study, to clarify the chemical structures of phytotoxic substances from *S. rostratum*, we isolated phenylpropanoid amides from the plant. Their structures were identified by comprehensive HR-ESIMS, NMR and ECD data. And the inhibitory functions of isolated phenylpropanoid amides on one model plant (*Arabidopsis thaliana*) were also investigated. In addition, the action mechanisms of active phenylpropanoid amides were revealed by antioxidant-related enzymes [Catalase (CAT), Peroxidase (POD), Superoxide dismutase (SOD)] activities and corresponding molecular docking analyses.

**Results and Discussion:**

Phytochemical research on the whole plant of *S. rostratum* led to the isolation and identification of four new phenylpropanoid amides (1−4), together with two known analogues (5−6). All the compounds showed phytotoxic effects with varying levels on the seed germination and root elongation of one model plant (*Arabidopsis thaliana*), especially compound 2 and 4. Likewise, compounds 2 and 4 displayed potent inhibitory effects on antioxidant-related enzyme (POD). In addition, compounds 2 and 4 formed common conventional hydrogen bonds with residues Ala34 and Ser35 in POD revealed by molecular docking analyses. These findings not only helped to reveal the invasion mechanism of S. rostratum from the perspective of “novel weapons hypothesis”, but also opened up new ways for the exploitation and utilization of *S. rostratum*.

## Introduction

With the acceleration of world economic globalization and international trade liberalization, especially the rapid development of tourism, alien invasive plants have posed a great threat to the economy, ecology, social security and national interests of many countries in the world (Seebens et al., 2017; Bonnamour et al., 2021). In China, some representative invasive plants, such as *Ageratina adenophora*, *Alternanthera philoxeroides, Ambrosia artemisiifolia* and *Mikania micrantha*, caused immeasurable losses every year (Tu et al., 2021). Therefore, preventing the invasion of alien plants, containing their spread and harm, and achieving sustainable control of existing invasive plants have become a major national demand to ensure the coordinated development of social progress and nature. In order to effectively prevent and control the further damage of invasive plants, it is important to deeply understand and reveal the invasion mechanism of alien plants from the research level (Enders et al., 2020). In recent years, the “novel weapons hypothesis” has successfully explained the causes of some alien plant invasion, and has become a research hotspot (Bais, 2003). The hypothesis suggested that alien plants gain a competitive advantage by producing some new phytotoxic substances to inhibit the growth of native plants, thus achieving successful invasion (Callaway and Ridenour, 2004). These phytotoxic substances belong to the secondary metabolites of plants, including small molecules of phenols, organic acids, terpenoids and nitrogenous compounds (Macías et al., 2019). However, the chemical structures, inhibitory functions and action mechanisms of phytotoxic substances of most invasive plants remain unclear. Therefore, fully clarifying the contribution of phytotoxic substances in the process of alien plant invasion is not only of great significance to reveal the invasion mechanism of alien plants, but also to open up new ways for the exploitation and utilization of invasive plants.

*Solanum rostratum* Duna., also known as Huang Hua Ci Qie and Kan Sa Si Ji, is an annual malignant weed of Solanaceae. It originated from North America and has spread to sixteen countries and regions in Europe, Asia, Africa and Oceania. In China, *S. rostratum* was first found in Liaoning Province in 1981. Now it has spread to Xinjiang, Jilin, Hebei, Shanxi, Beijing and other places, and the spreading trend is still expanding (Zhong et al., 2009; Liu et al., 2021). The Ministry of Ecology and Environment of China has listed *S. rostratum* as the fourth batch of invasive quarantine species in 2016. The species reproduces rapidly and competes with native plants for sunlight, water and living space, seriously damaging the ecological environment and biodiversity of invasion area. In addition, the species could produce a neurotoxin (solanine) that has a paralyzing effect on the central nervous system of human or livestock, and has become an extremely dangerous invasive plant (Wei et al., 1985; Wei et al., 2010). For a long time, the invasion mechanism of *S. rostratum* has been studied in many aspects, including its physiological characteristics (seed propagation, growth and development, pollination) and chemical defense against herbivorous insects (Karen, 1975; Eminniyaz et al., 2013; Liu et al., 2020; Liu et al., 2021; Yu et al., 2021). However, it is neglected to reveal the invasion mechanism of *S. rostratum* from the perspective of “novel weapons hypothesis”.

Previous studies showed that extracts from different parts of *S. rostratum* had certain inhibitory effects on seed germination and seedling growth of *Amaranthus retroflexus*, *Poa annua* and *Solanum lycopersicum* (Ping et al., 2012; Shao, 2015). However, these studies only focused on the phytotoxic effects of *S. rostratum* extracts, and the chemical structures, inhibitory functions and action mechanisms of its phytotoxic substances remain unclear. Like other higher plants, *S. rostratum* contains abundant secondary metabolites, mainly nitrogenous compounds, such as steroid alkaloids, pyrrole alkaloids, and phenylpropanoid amides (Novruzov et al., 1973; Liu et al., 2020; Liu et al., 2021). Among them, phenylpropanoid amides compounds are biosynthesized from cinnamic acid and phenylethylamine derivatives, which has complex chemical structures and diverse biological activities (Sun et al., 2015; Hao et al., 2022). A large number of studies have shown that phenylpropanoid amides have significant inhibitory effects on plant growth (Vishnoi et al., 2009; Thi et al., 2014; Thi et al., 2017), and we speculated that these compounds may be the potential phytotoxic substances for *S. rostratum.*

In this study, to clarify the chemical structures of phytotoxic substances from *S. rostratum*, we isolated and identified phenylpropanoid amides from the plant. And the inhibitory functions of isolated phenylpropanoid amides on one model plant (*Arabidopsis thaliana*) were also investigated. In addition, the action mechanisms of active phenylpropanoid amides were revealed by antioxidant-related enzymes [Catalase (CAT), Peroxidase (POD), Superoxide dismutase (SOD)] activities and corresponding molecular docking analyses.

## Materials and methods

### General

Column chromatography was carried out using silica gel (3.0 × 30 cm, Qingdao Marine, China), CHP20P (3.0 × 25 cm, Mitsubishi, Japan), octadecylsilyl silica (ODS) (YMC, Japan) and Sephadex LH-20 (1.5 × 180 cm, GE Healthcare, Sweden). Semi-preparative high performance liquid chromatography (HPLC) was conducted on a 1260 system (Agilent, USA) equipped with an XDB-C_18_ column (9.4 × 250 mm, 5 *μ*m, Agilent, USA). Ultra violet (UV) spectra were determined using a 241 spectrophotometer (Perkin Elmer, USA). High resolution electrosparay ionization mass spectrum (HR-ESIMS) spectra were recorded on a 6545 Q-TOF instrument (Agilent, USA). Nuclear magnetic resonance (NMR) spectra were acquired using an AV-600 instrument (Bruker, Germany). Electronic circular dichroism (ECD) spectra were obtained using a MOS-450 instrument (Bio-Logic, France). The solvents used in column chromatography were analytical grade (Tianjin Hengxing, China), the solvents used in HPLC, HR-ESIMS, ECD and activity assays were chromatographic grade (Sigma-Aldrich, USA), and the solvent used in NMR was methanol-*d*_4_ (Cambridge Isotope Laboratories, USA).

### Plant material

The whole plant of *S. rostratum* was collected from Baicheng, Jilin Province, China (123° 31′ E, 45° 98′ N) in September 2017 and identified by Professor Meini Shao (Shenyang Agricultural University). A voucher specimen (NO.ZW20170906) was deposited in the herbarium of Shenyang Agricultural University. The seeds of *A. thaliana* (Col-0) were obtained from Professor Xianling Wang (Shenyang Agricultural University) and stored in a 4°C refrigerator.

### Extraction and isolation

The air-dried *S. rostratum* (20 kg) was extracted with 70% ethanol (150 L × 2 times × 10 d, at room temperature). The concentrated extract (800 g) was successively partitioned with petroleum ether (8 L × 5 times) and ethyl acetate (8 L × 5 times). The ethyl acetate fraction (210 g) was subjected to silica gel column chromatography eluted with gradient dichloromethane: methanol (99: 1–50: 50, *v*/*v*) to obtain four subfractions (Fr. A–Fr. D). Fr. B (27 g) was subjected to silica gel column chromatography eluted with gradient dichloromethane: methanol (98: 2–50: 50, *v*/*v*) to obtain six subfractions (Fr. B-1–Fr. B-6). Fr. B-2 (3.5 g) was subjected to CHP20P column chromatography eluted with gradient methanol: H_2_O (10: 90–90: 10, *v*/*v*) to obtain five subfractions (Fr. B-2-1–Fr. B-2-5). Fr. B-2-2 (800 mg) was subjected to Sephadex LH-20 column chromatography eluted with isocratic methanol to yield four subfractions (Fr. B-2-2-1–Fr. B-2-2-4). Fr. B-2-2-1 (186 mg) was purified using semi-preparative HPLC (210 nm, 3.0 mL/min) eluted with isocratic methanol: H_2_O (15: 85, *v*/*v*) to obtain compounds 1 (3.5 mg, *t*_R_ = 47.5 min), 2 (8.4 mg, *t*_R_ = 32.1 min) and 3 (19.8 mg, *t*_R_ = 68.6 min), respectively, see **Figure S29**. Similarly, Fr. B-2-2-2 (172 mg) was purified using semi-preparative HPLC (210 nm, 3.0 mL/min) eluted with isocratic methanol: H_2_O (10: 90, *v*/*v*) to obtain compound 4 (4.6 mg, *t*_R_ = 37.2 min), see **Figure S29**. Fr. C (34 g) was subjected to silica gel column chromatography eluted with gradient dichloromethane: methanol (90: 10–50: 50, *v*/*v*) to obtain six subfractions (Fr. C-1–Fr. C-6). Fr. C-3 (4.1 g) was subjected to ODS column chromatography eluted with gradient methanol: H_2_O (10: 90–100: 0, *v*/*v*) to obtain seven subfractions (Fr. C-3-1–Fr. C-3-7). Fr. C-3-4 (1.2 g) was subjected to silica gel column chromatography eluted with gradient dichloromethane: methanol (90: 10–50: 50, *v*/*v*) to obtain seven subfractions (Fr. C-3-4-1–Fr. C-3-4-7). Fr. C-3-4-7 (67 mg) was purified using semi-preparative HPLC (210 nm, 5.0 mL/min) eluted with isocratic methanol: H_2_O (15: 85, *v*/*v*) to obtain compounds 5 (3.2 mg, *t*_R_ = 28.5 min) and 6 (2.2 mg, *t*_R_ = 21.4 min), respectively, see **Figure S29**. All the obtained compounds (1−6) were dried by a drying incubator and stored in a 4°C refrigerator.

Phenylpropylcyclamide A (1): yellowish brown oil; ECD (methanol) *λ*_max_ (△*ε*) 217 (-11.96), 227 (+13.90), 301 (-21.65) nm; HR-ESIMS at *m/z* 304.0947 [M+Na]^+^ (calcd for C_17_H_15_NO_3_Na, 304.0950); ^1^H and ^13^C NMR data, see **Table 1**.

**Table 1:** ^1^H (600 MHz) and ^13^C NMR (150 MHz) data of compounds 1−4 in methanol-*d*_4_.

position	1		2		3		4	
	*δ*_C_	*δ*_H_	*δ*_C_	*δ*_H_	*δ*_C_	*δ*_H_	*δ*_C_	*δ*_H_
2	175.3		167.3		167.5		167.7	
3	130.6		130.2		129.6		129.1	
4	43.7	4.50 (1H, brd, 7.8)	115.1		115.6		115.4	
5	51.1	3.90 (1H, dd, 9.6, 7.8) 3.21 (1H, dd, 9.6, 1.5)	122.6		122.3		122.5	
6	134.2	7.38 (1H, s)	132.8	7.81 (1H, s)	133.4	7.83 (1H, s)	134.5	7.86 (1H, s)
5-CH_3_			14.8	2.26 (3H, s)	14.6	2.27 (3H, s)	15.1	2.30 (3H, s)
1′	126.4		124.1		123.7		127.6	
2′	133.3	7.19 (1H, d, 8.6)	133.5	7.48 (1H, d, 8.5)	133.9	7.59 (1H, d, 8.6)	114.6	7.02 (1H, d, 2.1)
3′	116.9	6.61 (1H, d, 8.6)	116.7	6.58 (1H, d, 8.5)	118.2	7.01 (1H, d, 8.6)	145.4	
4′	161.5		161.4		159.3		148.5	
5′	116.9	6.61 (1H, d, 8.6)	116.7	6.58 (1H, d, 8.5)	118.2	7.01 (1H, d, 8.6)	115.3	6.75 (1H, d, 8.6)
6′	133.3	7.19 (1H, d, 8.6)	133.5	7.48 (1H, d, 8.5)	133.9	7.59 (1H, d, 8.6)	123.7	6.93 (1H. dd, 8.6, 2.1)
4′-OCH_3_					55.7	3.82 (3H, s)		
1′′	135.1		127.0		127.1		127.2	
2′′	129.0	7.05 (1H, d, 8.5)	130.6	7.36 (1H, d, 8.6)	130.6	7.35 (1H, d, 8.7)	130.7	7.37 (1H, d, 8.5)
3′′	116.7	6.70 (1H, d, 8.5)	116.5	6.67 (1H, d, 8.6)	116.7	6.65 (1H, d, 8.7)	116.8	6.64 (1H, d, 8.5)
4′′	157.5		158.6		158.7		158.6	
5′′	116.7	6.70 (1H, d, 8.5)	116.5	6.67 (1H, d, 8.6)	116.7	6.65 (1H, d, 8.7)	116.8	6.64 (1H, d, 8.5)
6′′	129.0	7.05 (1H, d, 8.5)	130.6	7.36 (1H, d, 8.6)	130.6	7.35 (1H, d, 8.7)	130.7	7.37 (1H, d, 8.5)

Phenylpropylcyclamide B (2): yellowish brown oil; HR-ESIMS at *m/z* 316.0953 [M+Na]^+^ (calcd for C_18_H_15_NO_3_Na, 316.0950); ^1^H and ^13^C NMR data, see **Table 1**.

Phenylpropylcyclamide C (3): yellowish brown oil; HR-ESIMS at *m/z* 330.1109 [M+Na]^+^ (calcd for C_19_H_17_NO_3_Na, 330.1106); ^1^H and ^13^C NMR data, see **Table 1**.

Phenylpropylcyclamide D (4): yellowish brown oil; HR-ESIMS at *m/z* 332.0873 [M+Na]^+^ (calcd for C_18_H_15_NO_4_Na, 332.0899); ^1^H and ^13^C NMR data, see **Table 1**.

*N-trans-p*-coumaroyl-3′,4′-dihydroxyphenylethylamine (5): yellowish amorphous powder; ^1^H and ^13^C NMR data, see **Figures S25, S26**.

*trans-N*-coumaroyltyramine (6): yellowish amorphous powder; ^1^H and ^13^C NMR data, see **Figures S27, S28**.

### ECD calculations

The ECD calculations were performed as a previously described method with little modifications (Liu et al., 2021). Firstly, the conformational searches were carried out using Spartan 14.0 program under MMFF94 force field. Then, the possible conformations were optimized using Gaussian 09 program at the B3LYP/6-31G (d) level. Subsequently, the theoretical calculations of ECD were calculated using time-dependent density functional theory (TDDFT) at the B3LYP/6-311 + G (2d, p) level in methanol. Finally, the ECD curves were obtained using SpecDis 1.60 program based on the Boltzmann weighting of each conformation.

### Seed germination bioassay

The seed germination assay was performed as a previously described method with little modifications (Liu et al., 2023). Firstly, *A. thaliana* seeds were sterilized with ethanol: H_2_O (75: 25, *v*/*v*) and rinsed with distilled water (500 mL × 3 times × 2 min), respectively. The sterilized seeds were stored in a 4°C refrigerator for 3 d before use. Then, the measured compounds were dissolved with methanol and added to different volumes of 1/2 MS medium, respectively, to obtain the medium containing different concentrations of compounds (100 *µ*g/mL, 50 *µ*g/mL, and 25 *µ*g/mL). The medium with the same volume of methanol was used as blank control. A sulfonylurea herbicide (Triasulfuron) (100 *µ*g/mL, 50 *µ*g/mL, and 25 *µ*g/mL) was used as positive control. Subsequently, the 3.0 mL of medium was transferred to each of the 6 cm petri dishes, respectively. After the medium was naturally cooled to a solid state, thirty sterilized seeds were evenly arranged and cultured in an illumination incubator (day 25°C 16 h, night 20°C 8 h, light intensity 100 *µ*M m^-2^ s^-1^). Finally, the number of germinated seeds were checked when the most of blank control seeds (≥ 90%) had germinated (three replicates each concentration). Inhibitory rate (%) was calculated as (Sc-S_T_)/Sc × 100 (Sc, the number of germinated seeds of blank control; S_T_, the number of germinated seeds of treatment).

### Root elongation assay

The root elongation assay was performed as a previously described method with little modifications (Liu et al., 2022). Firstly, *A. thaliana* seeds and the medium containing different concentrations of compounds were pretreated as described above. Then, the 1.5 mL of medium was transferred to each of the 6-well plates, respectively. Subsequently, after the medium was naturally cooled to a solid state, ten sterilized seeds were evenly arranged in a row and cultured vertically in an illumination incubator (day 25°C 16 h, night 20°C 8 h, light intensity 100 *µ*M m^-2^ s^-1^). Finally, the roots lengths were measured when the roots of blank control reached the bottom of the 6-well plate (three replicates each concentration). Inhibitory rate (%) was calculated as (Rc-R_T_)/Rc × 100 (Rc, the average lengths of blank control; R_T_, the average lengths of treatment).

### Antioxidant-related enzymes assay

The extraction of enzymes was performed as a previously described method with little modifications (Chen et al., 2009). Firstly, *A. thaliana* seeds and the medium containing 100 *μ*g/mL of compounds 2 and 4 were pretreated as described above. Then, the 1.5 mL of medium was transferred to each of the 6-well plates, respectively. After the medium was naturally cooled to a solid state, ten sterilized seeds were evenly arranged in a row and cultured vertically in an illumination incubator (day 25°C 16 h, night 20°C 8 h, light intensity 100 *µ*M m^-2^ s^-1^). Subsequently, the roots lengths were measured when the roots of blank control reached the bottom of the 6-well plate. After measuring root length, we weighed 0.2 g of *A. thaliana* for antioxidant-related enzymes assay. Finally, the treated *A. thaliana* (0.2 g) were homogenized with 5.0 mL phosphate buffer saline (PBS) (0.05 M, pH = 7.0) in cold mortar. The mixture was centrifuged at 12,000 rpm for 10 min at 4°C, and the supernatant was used for the antioxidant-related enzymes assay. The same volume of PBS was used as blank control.

The CAT assay was performed as ultraviolet absorption method with little modifications (He et al., 2009). The 0.1 mL enzyme solution was mixed with 1.0 mL H_2_O_2_ (0.2 mM, pH = 7.0) and 3.9 mL PBS (0.05 M, pH = 7.0). The absorbance of mixture was measured at 240 nm. One unit of CAT activity was defined as a decrease in absorbance of 0.1 per milligram of protein per minute. The POD assay was performed as guaiacol method with little modifications (Chen et al., 2009). The 0.1 mL enzyme solution was mixed with 1.0 mL H_2_O_2_ (0.2 mM, pH = 7.0), 2.9 mL PBS (0.05 M, pH = 7.0), and 1.0 mL guaiacol (0.05 M). The absorbance of mixture was measured at 470 nm. One unit of POD activity was defined as an increase in absorbance of 0.1 per milligram of protein per minute. The SOD assay was performed as nitro blue tetrazolium (NBT) method with little modifications (Chen et al., 2009). The 0.1 mL enzyme solution was mixed with 2.9 mL PBS (0.05 M, pH = 7.0) containing 2.0 *µ*M riboflavin, 75.0 *µ*M NBT, 10.0 *µ*M ethylenediaminetetraacetic acid, and 13.0 *µ*M methionine. The mixture was irradiated by fluorescent lamp (4000 lx) for 20 min. The absorbance was measured at 560 nm. One unit of SOD activity was defined as the amount of enzyme required to inhibit the reduction of NBT by 50% per milligram of protein. The above experiments were repeated three times.

### Molecular docking analyses

Firstly, the structure of receptor (POD) was obtained from RCSB PDB (https://www.rcsb.org/) with code: 1PA2 (Ostergaard et al., 2000) and further optimized by SYBYL-X 2.0 program. Then, the structures of ligands (compouds 2 and 4) were optimized by the ChemDraw-3D 14.0 program. Subsequently, an active pocket centered on x (1.42), y (35.11), z (34.72) was created based on the binding sites of the original ligand and protein. Finally, the Molegro Virtual Docker 4.0 program was performed for the docking studies.

### Statistical analyses

All data were presented as means ± SD of three independent experiments. Statistical comparisons between group means were performed using One-way ANOVA. A *p* value < 0.05 was considered statistically significant.

## Results and discussion

**Figure 1:**
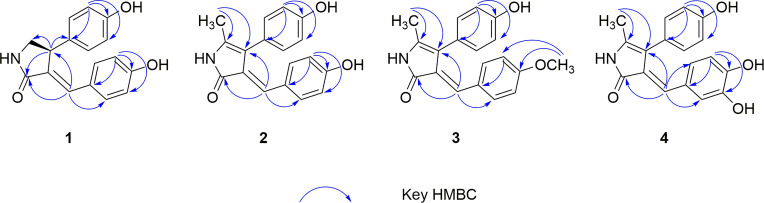
Key HMBC correlations of compounds 1−4.

**Figure 2:**
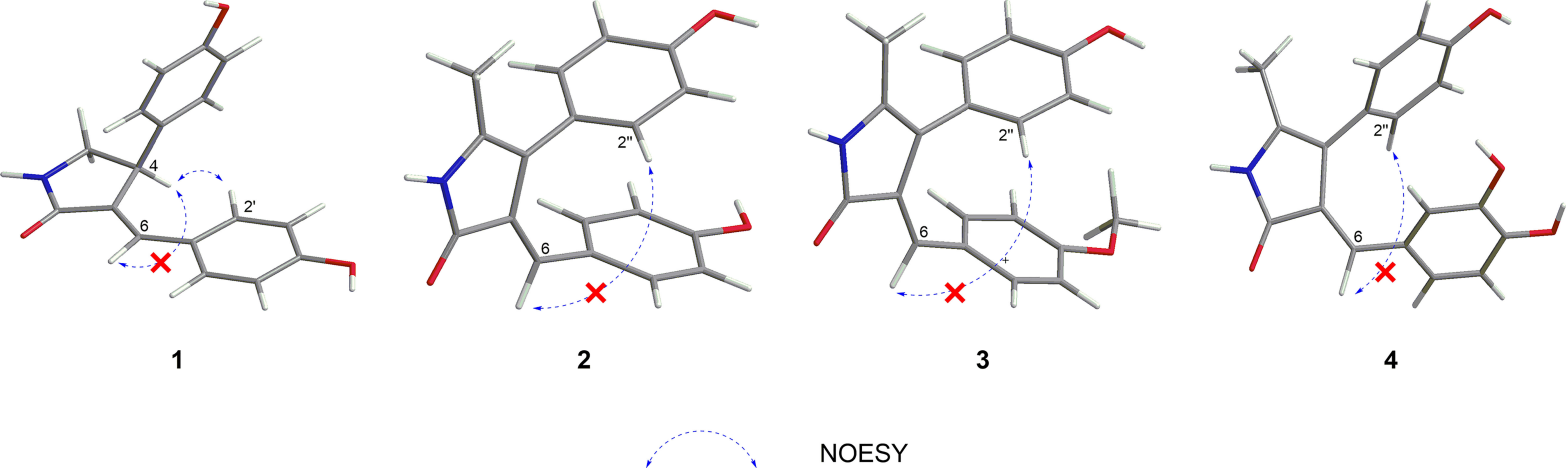
Key NOESY correlations of compounds 1−4.

**Figure 3:**
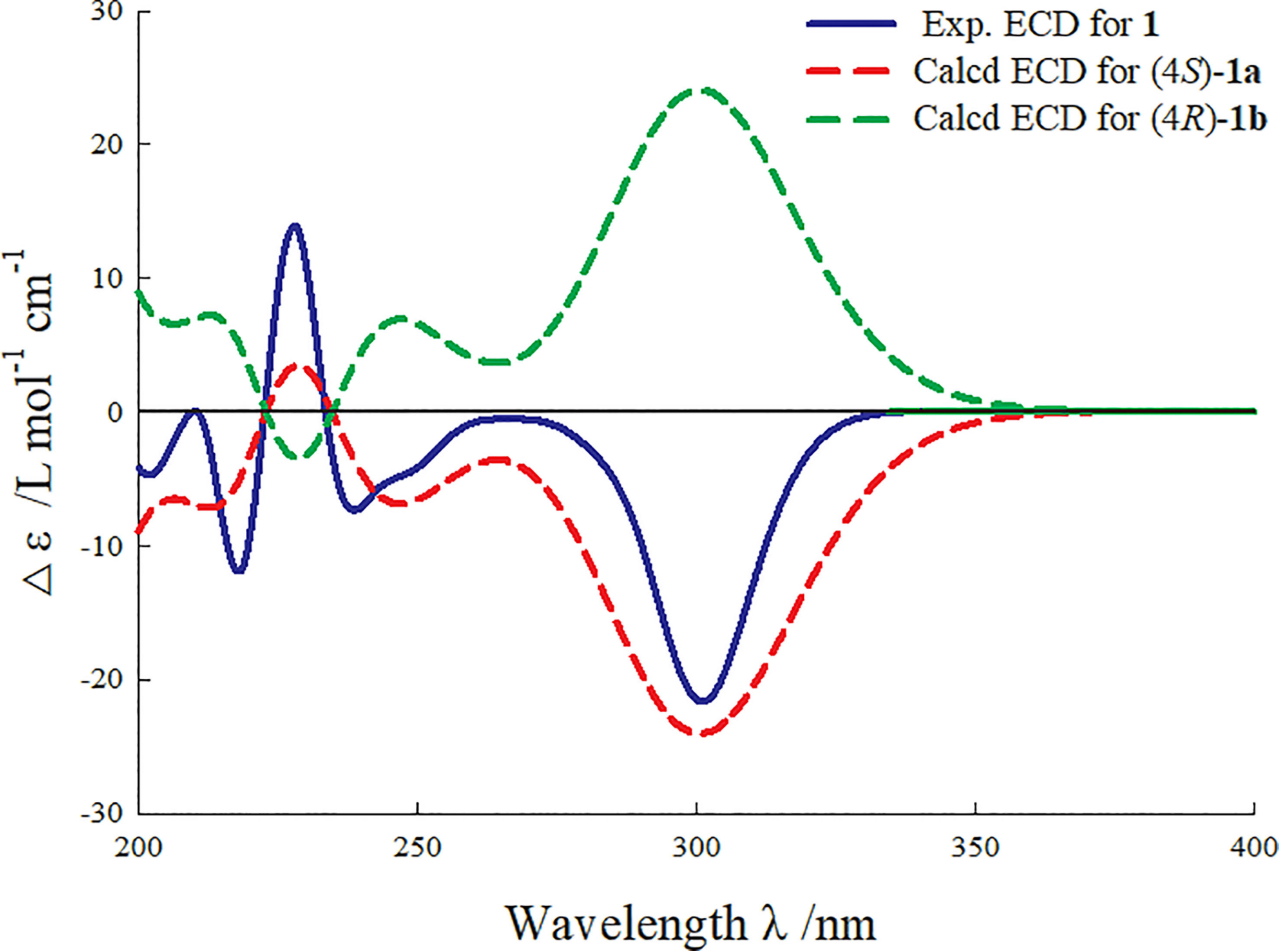
Experimental and calculated ECD curve of compound 1.

**Figure 4:**
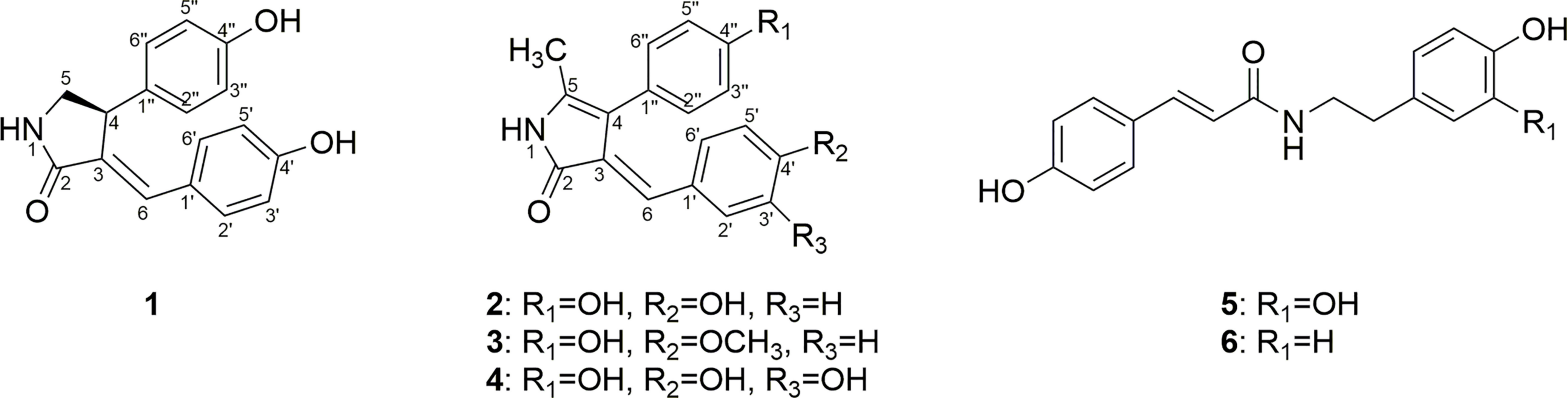
Structures of compounds 1−6.

Phenylpropylcyclamide A (1) was isolated as yellowish brown oil with C_17_H_15_NO_3_ (eleven degrees of unsaturation) according to HR-ESIMS data (*m/z* 304.0947 [M+Na]^+^, calcd for C_17_H_15_NO_3_Na, 304.0950). Its 1D-NMR spectra (**Table 1**) showed one carbonyl group [*δ*_C_ 175.3 (C-2)], one double bond group [*δ*_H_ 7.38 (1H, s, H-6); *δ*_C_ 134.2 (C-6), 130.6 (C-3)], two *para*-oxy-substituted phenyl groups [*δ*_H_ 7.19 (2H, d, *J* = 8.6 Hz, H-2′, H-6′), 7.05 (2H, d, *J* = 8.5 Hz, H-2′′, H-6′′), 6.70 (2H, d, *J* = 8.5 Hz, H-3′′, H-5′′, 6.61 (2H, d, *J* = 8.6 Hz, H-3′, H-5′); *δ*_C_ 161.5 (C-4′), 157.5 (C-4′′), 135.1 (C-1′′), 133.3 (C-2′, C-6′), 129.0 (C-2′′, C-6′′), 126.4 (C-1′), 116.9 (C-3′, C-5′), 116.7 (C-3′′, C-5′′)], respectively. Comparison of its 1D NMR spectra with a known compound Tamaractam showed that they were very similar except for two missing methoxy groups (Yao et al., 2017), which indicated that 1 possessed a phenylpropanoid amides skeleton. In the heteronuclear multiple bond correlation (HMBC) spectrum (**Figure 1**), the key correlations of H-4/C-2, C-5, C-6′′; H-6/C-2, C-4, C-2′; H-5′/C-1′, C-3′, C-4′; H-5′′/C-1′′, C-3′′, C-4′′ established the planar structure of compound 1. In the nuclear overhauser and exchange spectroscopy (NOESY) spectrum (**Figure 2**), the key correlation of H-4/H-2′ and the lack of correlation of H-4/H-6 indicated that the configuration of double bond between C-3 and C-6 was *E.* Furthermore, the computed ECD curve of (4*S*)-1a matched well with the experimental result (**Figure 3**), which established the stereoscopic structure of compound 1. Thus, the structure of 1 was identified and named Phenylpropylcyclamide A (**Figure 4**).

Phenylpropylcyclamide B (2) was isolated as yellowish brown oil with C_18_H_15_NO_3_ (twelve degrees of unsaturation) according to HR-ESIMS data (*m/z* 316.0953 [M+Na]^+^, calcd for C_18_H_15_NO_3_Na, 316.0950). Comparison of its 1D NMR spectra with compound 1 showed that they were very similar except for one additional methyl group [*δ*_H_ 2.26 (3H, s, 5-CH_3_); *δ*_C_ 14.8 (5-CH_3_)] and one additional double bond group [*δ*_C_ 122.6 (C-5), 115.1 (C-4)], which indicated that 2 also possessed a phenylpropanoid amides skeleton. In the HMBC spectrum (**Figure 1**), the key correlations of H-5-CH_3_/C-4, C-5; H-6/C-2, C-4 revealed that the methyl group was connected to Phenylpropylcyclamide A by C-5 and the double bond group formed between C-4 and C-5. In the NOESY spectrum (**Figure 2**), the lack of correlation of H-6/H-2′′ indicated that the configuration of double bond between C-3 and C-6 was *E.* Thus, the structure of 2 was identified and named Phenylpropylcyclamide B (**Figure 4**).

Phenylpropylcyclamide C (3) was isolated as yellowish brown oil with C_19_H_17_NO_3_ (twelve degrees of unsaturation) according to HR-ESIMS data (*m/z* 330.1109 [M+Na]^+^, calcd for C_19_H_17_NO_3_Na, 330.1106). Comparison of its 1D NMR spectra with compound 2 showed that they were very similar except for one additional methoxy group [*δ*_H_ 3.82 (3H, s, 4′-OCH_3_); *δ*_C_ 55.7 (4′-OCH_3_)], which indicated that 3 also possessed a phenylpropanoid amides skeleton. In the HMBC spectrum (**Figure 1**), the key correlations of H-4′-OCH_3_/C-4′, C-5′ revealed that the methoxy group was connected to Phenylpropylcyclamide B by C-4′. In the NOESY spectrum (**Figure 2**), the lack of correlation of H-6/H-2′′ indicated that the configuration of double bond between C-3 and C-6 was *E.* Thus, the structure of 3 was identified and named Phenylpropylcyclamide C (**Figure 4**).

Phenylpropylcyclamide D (4) was isolated as yellowish brown oil with C_18_H_15_NO_4_ (twelve degrees of unsaturation) according to HR-ESIMS data (*m/z* 332.0873 [M+Na]^+^, calcd for C_18_H_15_NO_4_Na, 332.0899). Comparison of its 1D NMR spectra with compound 2 showed that they were very similar except for one additional 3, 4-dioxy-substituted phenyl group [*δ*_H_ 7.02 (1H, d, *J* = 2.1 Hz, H-2′), 6.93 (1H, dd, *J* = 8.6, 2.1 Hz, H-6′), 6.75 (1H, d, *J* = 8.6 Hz, H-5′); *δ*_C_ 148.5 (C-4′), 145.3 (C-3′), 127.6 (C-1′), 123.7 (C-6′), 115.3 (C-5′), 114.5 (C-2′)] and one missing *para*-oxy-substituted phenyl group, which indicated that 4 also possessed a phenylpropanoid amides skeleton. In the HMBC spectrum (**Figure 1**), the key correlations of H-6/C-2, C-4, C-2′, C-6′ revealed that the 3, 4-dioxy-substituted phenyl group was connected to Phenylpropylcyclamide B by C-6. In the NOESY spectrum (**Figure 2**), the lack of correlation of H-6/H-2′′ indicated that the configuration of double bond between C-3 and C-6 was *E.* Thus, the structure of 4 was identified and named Phenylpropylcyclamide D (**Figure 4**).

The two other compounds 5 and 6 were known and identified as *N-trans-p*-coumaroyl-3′,4′-dihydroxyphenylethylamine and *trans-N*-coumaroyltyramine, respectively, based on the NMR data and literature comparison (Liu et al., 2020).

**Figure 5:**
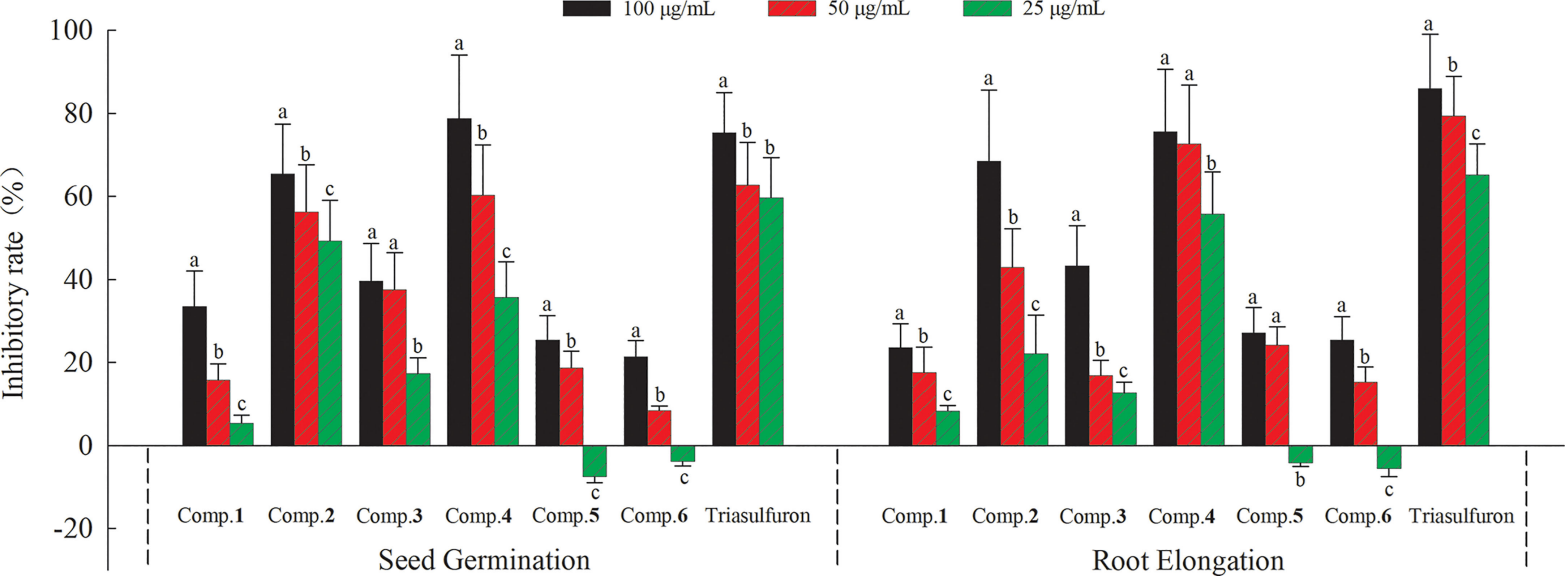
Phytotoxic effects of compounds 1−6 on the seed germination and root elongation of *A. thaliana*. Different letters indicate significant differences between concentration treatments (*p* < 0.05).

A large number of studies have shown that phenylpropanoid amides have significant inhibitory effects on plant growth (Vishnoi et al., 2009; Thi et al., 2014; Thi et al., 2017), and we speculated that these compounds may be the potential phytotoxic substances for *S. rostratum.* In addition, *A. thaliana* is an important model plant for evaluating phytotoxic effects, which has been reported in many literatures (Liu et al., 2013; Chen et al., 2016; Li et al., 2019). Therefore, phenylpropanoid amides (1−6) were then assessed for their phytotoxic effects on the seed germination and root elongation of *A. thaliana*. As shown in **Figure 5**, these compounds showed phytotoxic effects with varying levels. Compounds 1, 3, 5 and 6 exhibited weak to moderate phytotoxic effects at 100 *μ*g/mL with seed germination and root elongation inhibitory rates ranged from 21.37 to 40.05% and 23.56 to 43.31%, respectively. Among them, compounds 5 and 6 had slight promotion effects on the seed germination and root elongation of *A. thaliana* at 25 *μ*g/mL. On the whole, compounds 2 and 4 exhibited stronger phytotoxic effects compared with other compounds. It is notable that compound 4 exhibited potent phytotoxic effects at 100 *μ*g/mL (78.69% on seed germination and 75.56% on root elongation), which even comparable to Triasulfuron (75.32% on seed germination and 82.98% on root elongation).

**Figure 6:**
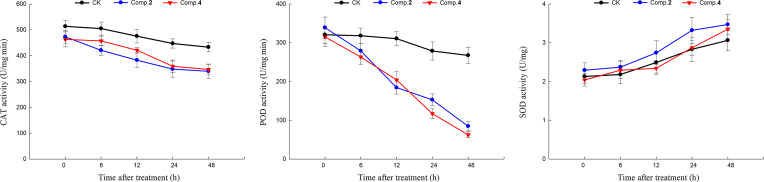
The effects of compounds 2 and 4 (100 *μ*g/mL) on antioxidant-related enzymes (CAT, POD, SOD) activities of *A. thaliana*.

Plants could produce reactive oxygen species (ROS) when subjected to biological or abiotic stress. The accumulation of ROS could induce membrane injury and lipid peroxidation to damage cell structure, thus inhibiting plant growth. Antioxidant-related enzymes (CAT, POD and SOD) could effectively prevent the accumulation of high concentration ROS to reduce plant damage (Arora et al., 2002). Therefore, the action mechanisms of active phenylpropanoid amides (2 and 4) were revealed by antioxidant-related enzymes activities. The effects of compounds 2 and 4 on CAT, POD and SOD activities of *A. thaliana* at different time were shown in **Figure 6**. As a whole, the CAT and POD activities of compounds (2 and 4) and blank control treatment decreased with the extension of time, and the SOD activities of compounds (2 and 4) and blank control treatment increased with the extension of time. It is notable that POD activities decreased significantly by 40.63%, 45.23% and 68.40% under compound 2 treatment, and 34.27%, 58.04% and 76.68% under compound 4 treatment compared with the blank control at 12, 24 and 48 h, respectively, indicating that compounds 2 and 4 could inhibit plant growth probably by inhibiting POD enzyme effectively. It is appeared that POD may be a key target for compounds 2 and 4 to exert their phytotoxic effects.

**Figure 7:**
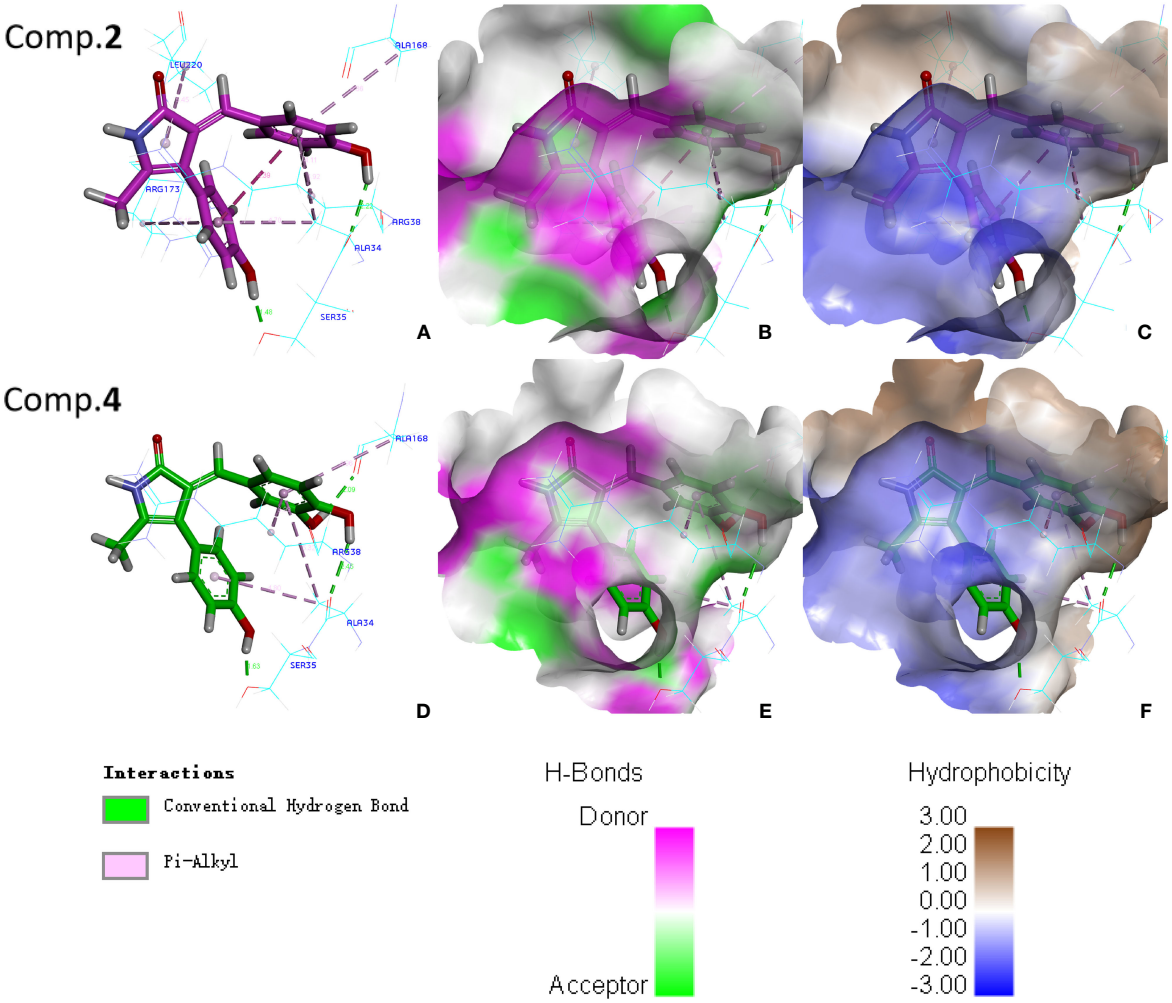
Molecular docking analyses of compounds 2 and 4 with POD: **(A)** Interactions between compound 2 and amino acid residues of POD; **(B)** H-bonds between compound 2 and active pocket of POD; **(C)** Hydrophobic property between compound 2 and active pocket of POD; **(D)** Interactions between compound 4 and amino acid residues of POD; **(E)** H-bonds between compound 4 and active pocket of POD; **(F)** Hydrophobic property between compound 4 and active pocket of POD.

It is well known that molecular docking provides visual analyses to explore the binding sites and modes of action of protein and ligand (Ferreira et al., 2015). In addition, molecular docking is generally meaningful only when combined with actual enzyme activity experiments. However, after testing the activities, there were no enough compounds 1, 5 and 6 to measure antioxidant-related enzymes assay. Compounds 2 and 4 were finally selected for molecular docking because of their significant effects on seed germination, root elongation and POD enzyme activities of *Arabidopsis thaliana*. As shown in **Table S1**, compound 4 (MolDock scores of −135.462 kcal/mol) had a lower binding energy with POD than compound 2 (MolDock scores of −128.591 kcal/mol), indicating that compound 4 binds to POD more stable than compound 2. It was also in good agreement with the phytotoxic activities and POD enzyme assay of compounds 2 and 4. In addition, phenolic hydroxyl groups and phenyl groups of compounds bound to neighboring amino acid residues though conventional hydrogen bonds and Pi-Alkyl interactions, respectively, indicating that the phenol structural fragment of the compounds 2 and 4 played a crucial role in their POD activities (**Figure 7**). On the other hand, studies have shown that conventional hydrogen bonds are more important than other interactions (Panigrahi and Desiraju, 2007). Compound 2 formed two conventional hydrogen bonds with residues Ala34 (distance 2.22 Å) and Ser35 (distance 1.48 Å) in POD. Meanwhile, compound 4 formed three conventional hydrogen bonds with residues Ala168 (distance 2.09 Å), Ala34 (distance 2.46 Å) and Ser35 (distance 1.63 Å) in POD (**Figure 7**). The above not only proved that more hydrogen bonds lead to more stable bonding of protein and ligand, but also suggested that the common residues Ala34 and Ser35 may be key active sites interacting with 2 and 4.

In recent years, alien invasive plants have posed a great threat to the economy, ecology, social security and national interests of many countries in the world (Seebens et al., 2017; Bonnamour et al., 2021). Studies have shown that the “novel weapons hypothesis” could successfully explain the causes of some alien plant invasion (Bais, 2003). The hypothesis suggested that alien plants gain a competitive advantage by producing some new phytotoxic substances that negatively affect on the growth of native plants (Callaway and Ridenour, 2004). Phenylpropanoid amides are the potential phytotoxic substances, which have significant inhibitory effects on plant growth (Vishnoi et al., 2009; Thi et al., 2014; Thi et al., 2017). In our study, four new phenylpropanoid amides (1−4), together with two known analogues (5−6) were isolated and identified from *S. rostratum.* Among them, compounds 2 and 4 showed potent phytotoxic effects on the seed germination and root elongation of *A. thaliana.* These two active compounds could inhibit plant growth probably by inhibiting POD enzyme effectively, thereby indirectly improving the competitive advantage of *S. rostratum*. We speculated that due to the lack of co-evolution between native and alien plants, antioxidant enzymes of *A. thaliana* have not adapted to the new compounds (2 and 4) from *S. rostratum*. However, further research of more native companion plants (non-model) is needed to confirm this. From another perspective, compounds 2 and 4 are expected to be the lead active substances with potential application of plant-derived herbicides. It will realize the transformation of *S. rostratum* waste into treasure, and also provide ideas for the exploitation and utilization of other invasive plants.

## Conclusion

In summary, four new phenylpropanoid amides (1−4), together with two known analogues (5−6) were isolated from the whole plant of *S. rostratum* and their structures were identified by HR-ESIMS, NMR and ECD data. All the compounds showed phytotoxic effects with varying levels on one model plant (*A. thaliana*), especially compound 2 and 4, which exhibited potent phytotoxic effects on the seed germination and root elongation at 100 *μ*g/mL. Likewise, compounds 2 and 4 displayed potent inhibitory effects on antioxidant-related enzyme (POD). In addition, compounds 2 and 4 formed common conventional hydrogen bonds with residues Ala34 and Ser35 in POD revealed by molecular docking analyses. Our findings not only helped to reveal the invasion mechanism of *S. rostratum* from the perspective of “novel weapons hypothesis”, but also opened up new ways for the exploitation and utilization of *S. rostratum.*

## Data availability statement

The original contributions presented in the study are included in the article/**Supplementary Material**, further inquiries can be directed to the corresponding author/s.

## Author contributions

ZXL designed the research and wrote the manuscript. XQM, NZ, LLY, HRY and LLZ performed the experiments and analyzed the data. TA and YBX reviewed and revised the draft. All authors contributed to the article and approved the submitted version.
