# Supplementary material for: Phenylpropanoid amides from Solanum rostratum and their phytotoxic activities against Arabidopsis thaliana
Source: Front Plant Sci. 2023 Apr 12;14:1174844. doi: 10.3389/fpls.2023.1174844 (PMC10130401; doi:10.3389/fpls.2023.1174844)
Supplement: Supplementary file 1 [file DataSheet_1.docx]

**Supporting Information**

**Phenylpropanoid amides from *Solanum rostratum* and their phytotoxic activities against *Arabidopsis thaliana***

Zhixiang Liu ^1^, Xiaoqing Ma ^1^, Nan Zhang ^1^, Linlin Yuan ^1^, Hongrui Yin ^1^, Tong An ^1,*^, Yubin Xu ^2,*^

^1^ College of Bioscience and Biotechnology, Shenyang Agricultural University, Shenyang 110866, P.R. China

^2^ Department of Pharmacy, Taizhou Central Hospital (Taizhou University Hospital), Taizhou 318000, P.R. China.

*Corresponding authors. Phone: +86-24-88487163. Fax: +86-24-88492799.

E-mail addresses: [fyl@syau.edu.cn](mailto:fyl@syau.edu.cn) (Y.-L. Feng); [xuyubin1988@126.com](mailto:xuyubin1988@126.com) (Y.-B. Xu).

**Figure S1.** The HR-ESIMS spectrum of compound **1**

**Figure S2.** The ^1^H-NMR spectrum of compound **1**

**Figure S3.** The ^13^C-NMR spectrum of compound **1**

**Figure S4.** The HSQC spectrum of compound **1**

**Figure S5.** The HMBC spectrum of compound **1**

**Figure S6.** The NOESY spectrum of compound **1**

**Figure S7.** The HR-ESIMS spectrum of compound **2**

**Figure S8.** The ^1^H-NMR spectrum of compound **2**

**Figure S9.** The ^13^C-NMR spectrum of compound **2**

**Figure S10.** The HSQC spectrum of compound **2**

**Figure S11.** The HMBC spectrum of compound **2**

**Figure S12.** The NOESY spectrum of compound **2**

**Figure S13.** The HR-ESIMS spectrum of compound **3**

**Figure S14.** The ^1^H-NMR spectrum of compound **3**

**Figure S15.** The ^13^C-NMR spectrum of compound **3**

**Figure S16** The HSQC spectrum of compound **3**

**Figure S17.** The HMBC spectrum of compound **3**

**Figure S18.** The NOESY spectrum of compound **3**

**Figure S19.** The HR-ESIMS spectrum of compound **4**

**Figure S20.** The ^1^H-NMR spectrum of compound **4**

**Figure S21.** The ^13^C-NMR spectrum of compound **4**

**Figure S22.** The HSQC spectrum of compound **4**

**Figure S23.** The HMBC spectrum of compound **4**

**Figure S24.** The NOESY spectrum of compound **4**

**Figure S25.** The ^1^H-NMR spectrum of compound **5**

**Figure S26.** The ^13^C-NMR spectrum of compound **5**

**Figure S27.** The ^1^H-NMR spectrum of compound **6**

**Figure S28.** The ^13^C-NMR spectrum of compound **6**

**Figure S29.** The flowchart of extraction and isolation

**Table S1**. Moldock scores of compounds with POD

**Figure S1.**


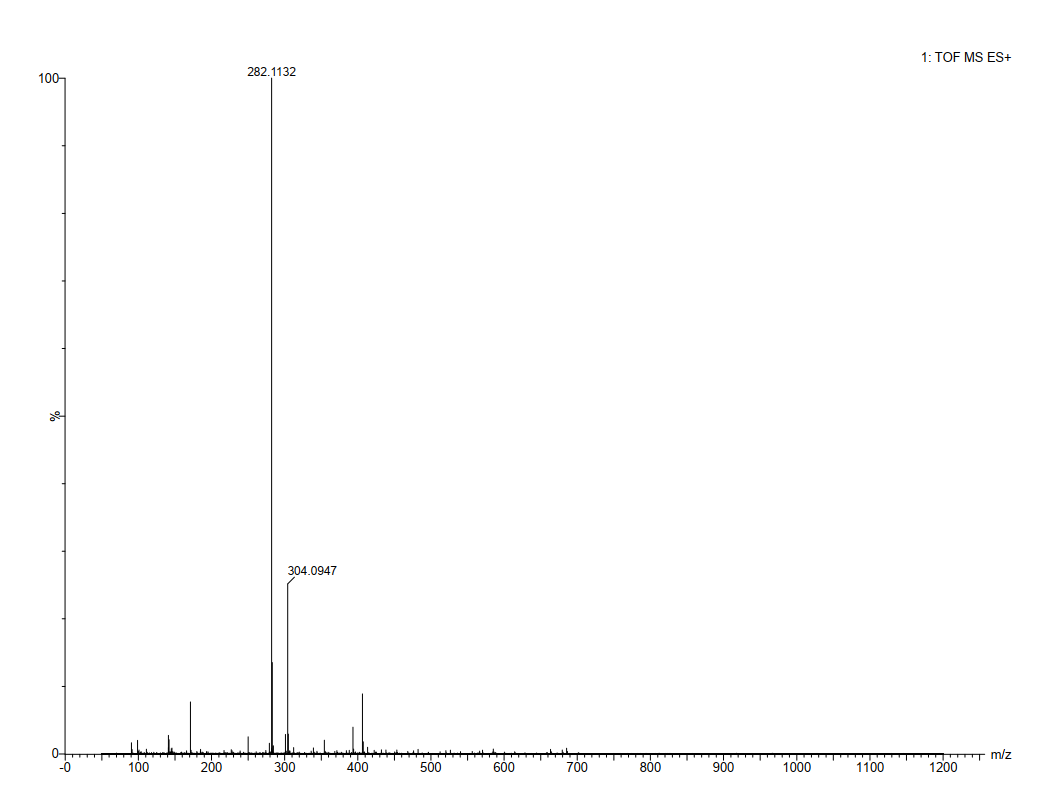


**Figure S2.**


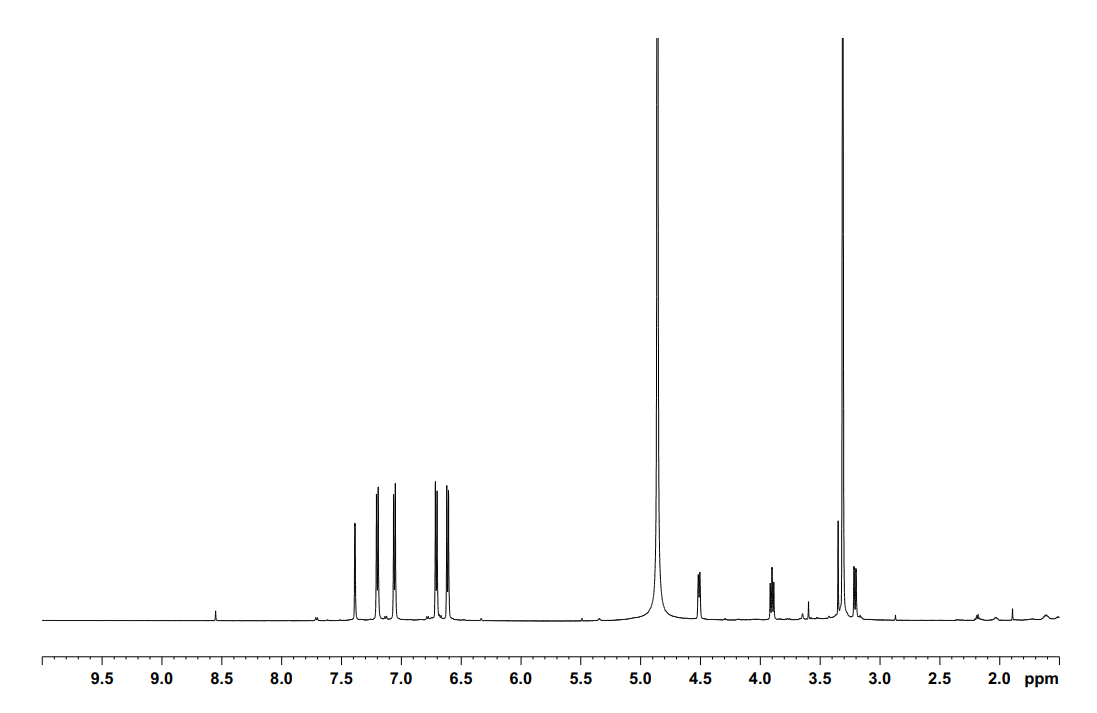


**Figure S3.**


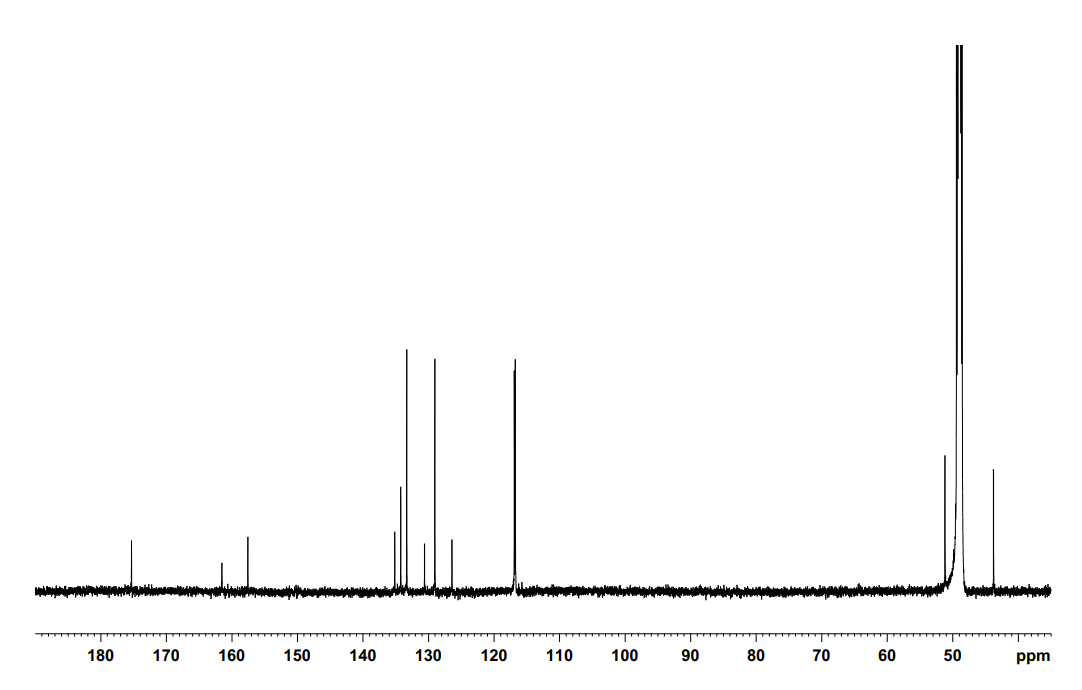


**Figure S4.**


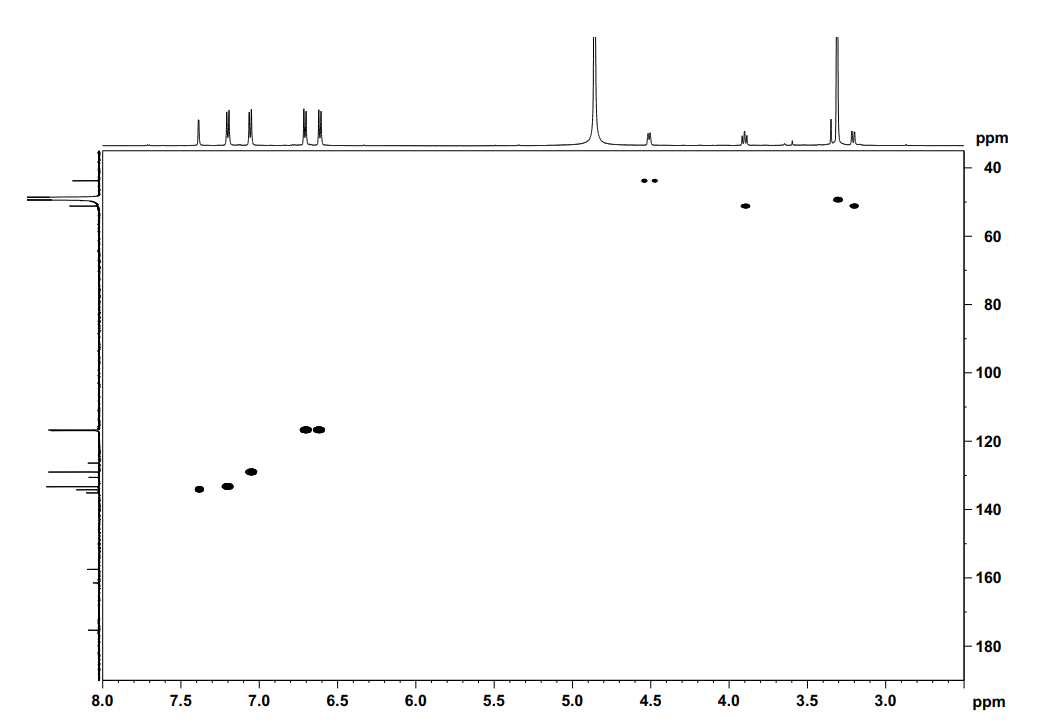


**Figure S5.**


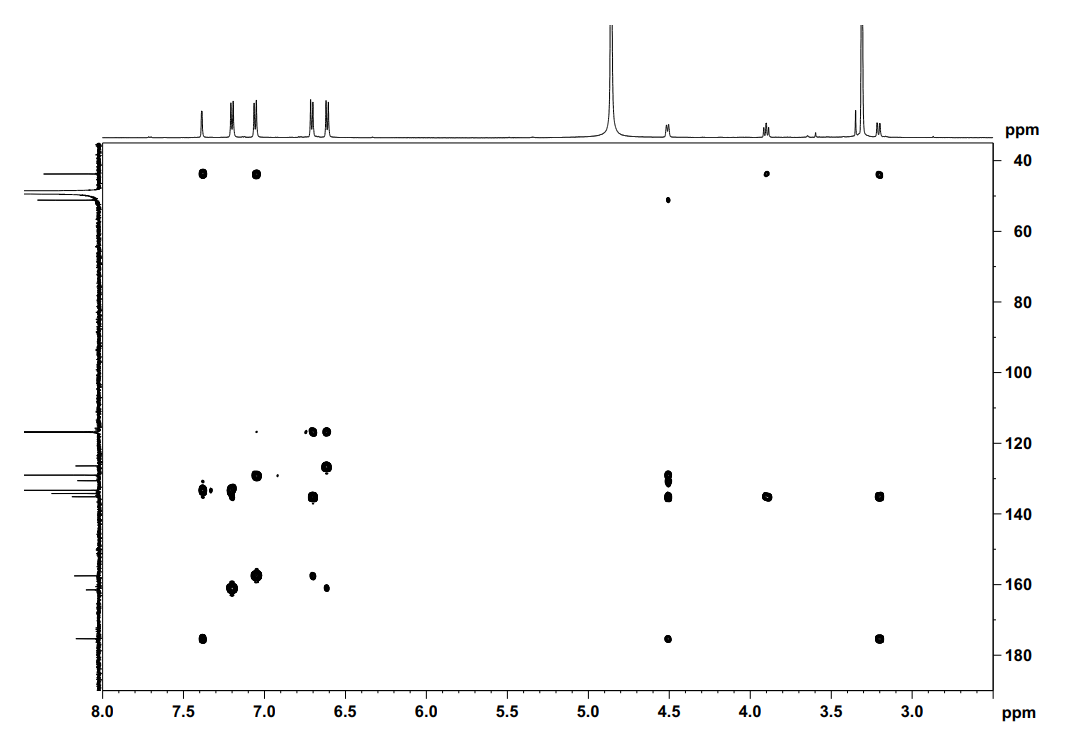


**Figure S6.**


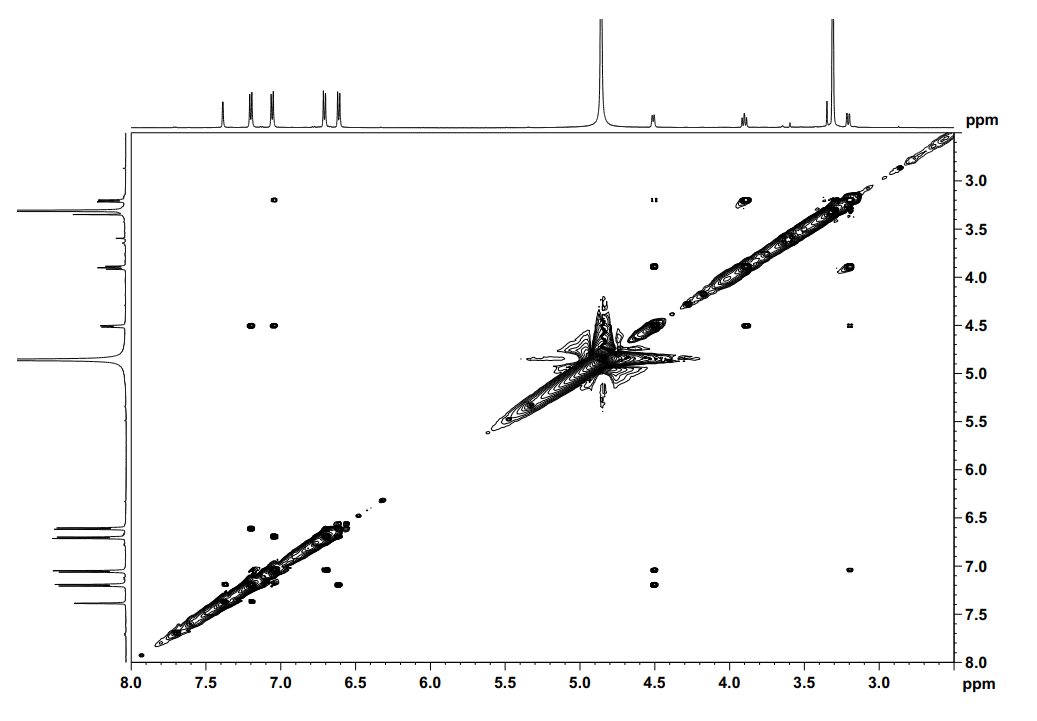


**Figure S7.**


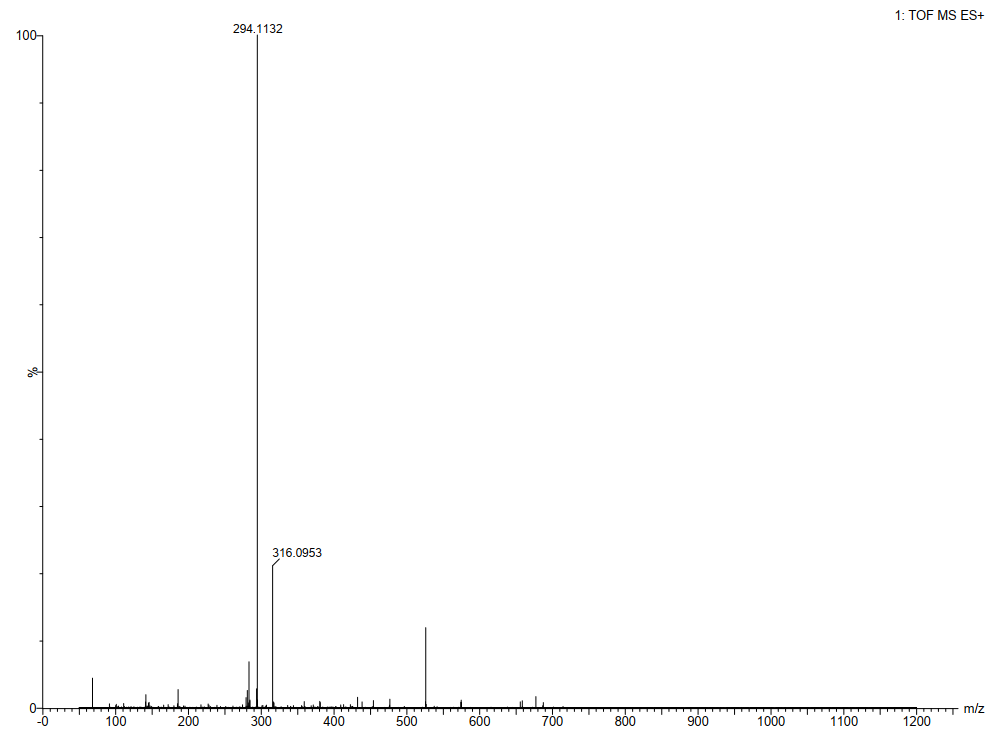


**Figure S8.**


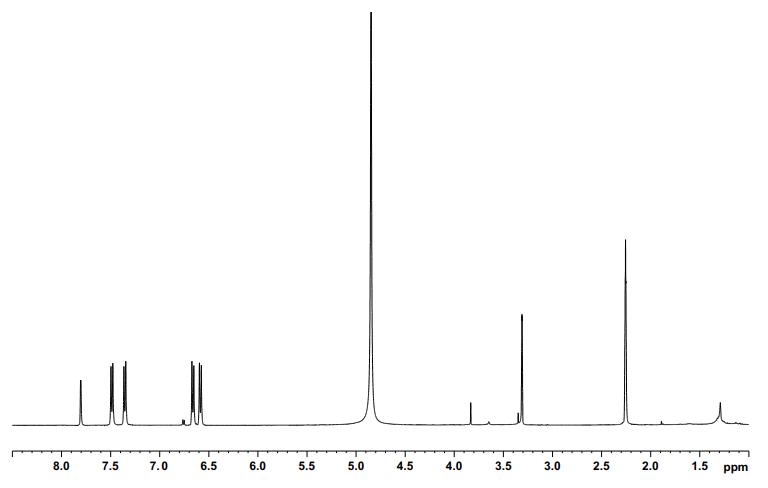


**Figure S9.**


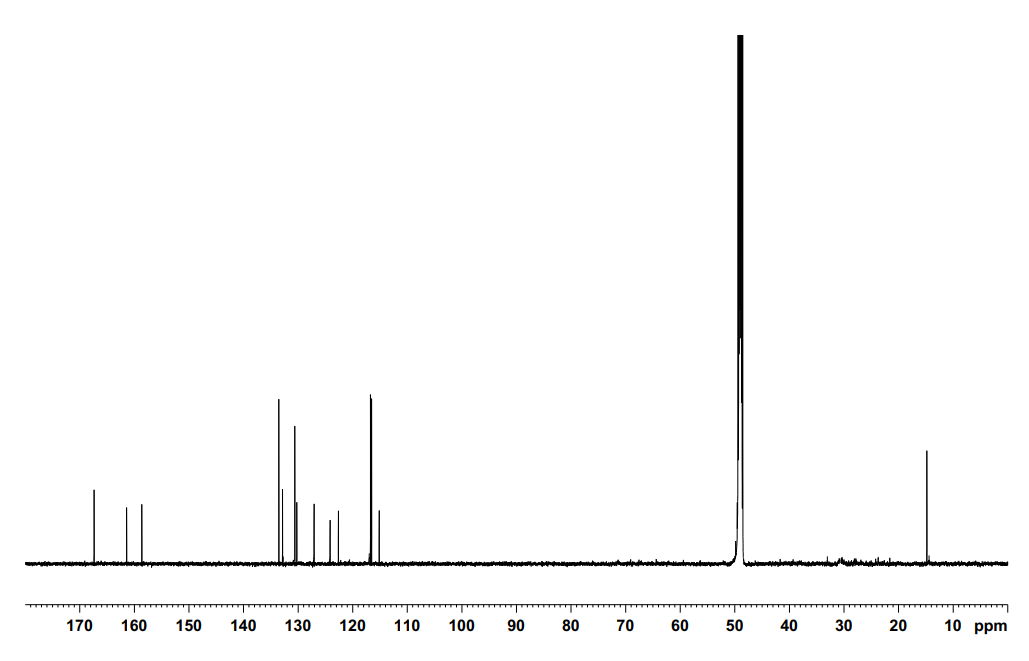


**Figure S10.**


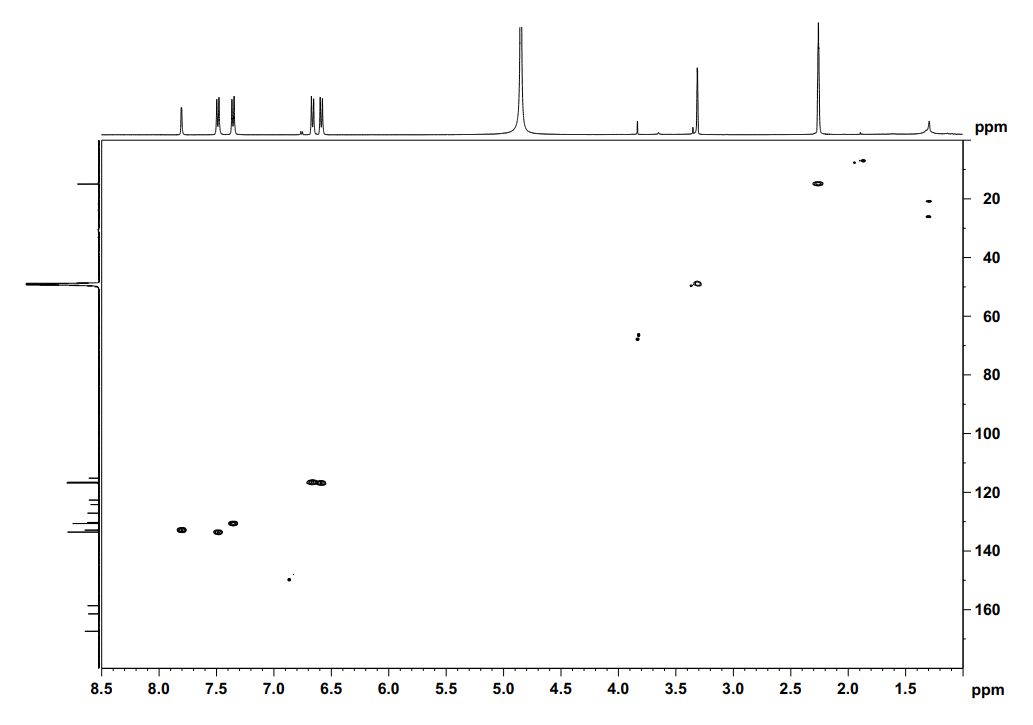


**Figure S11.**


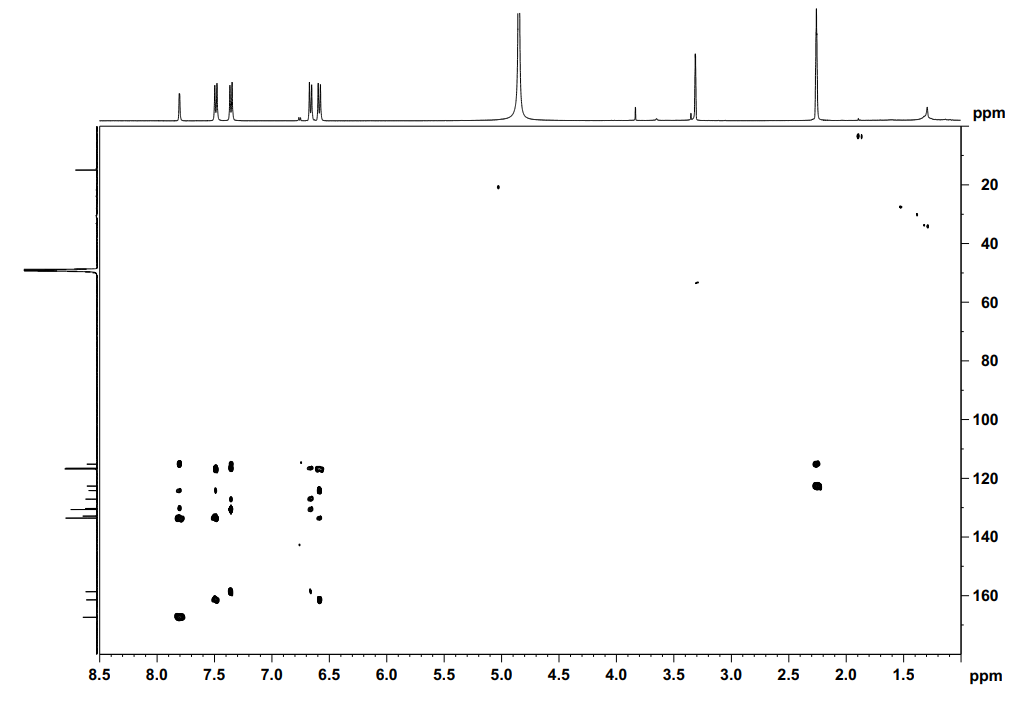


**Figure S12.**

.
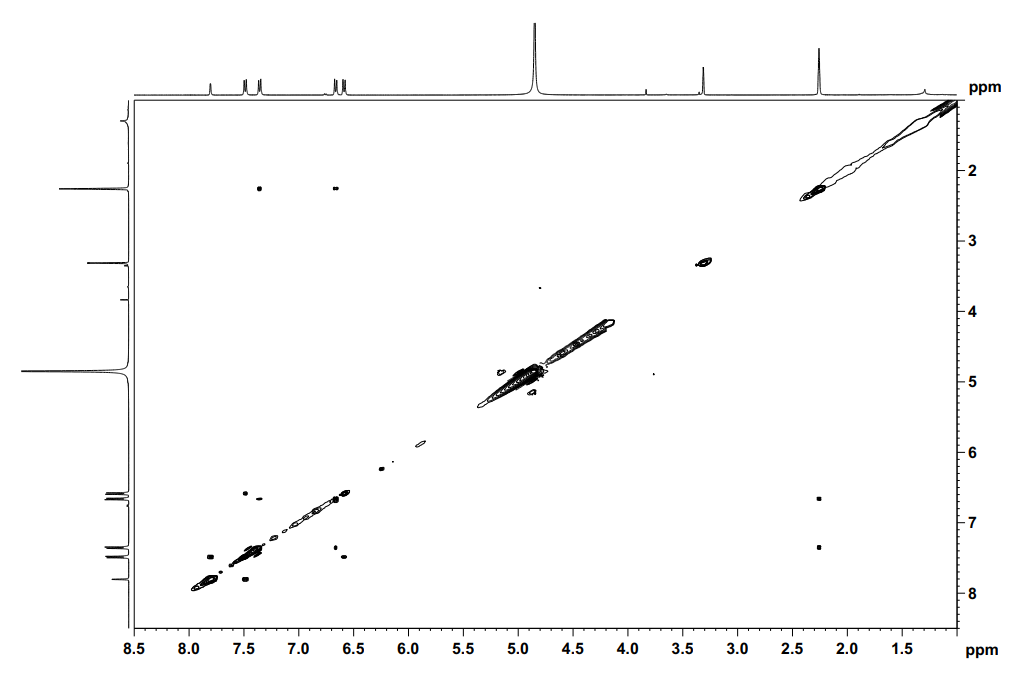


**Figure S13.**


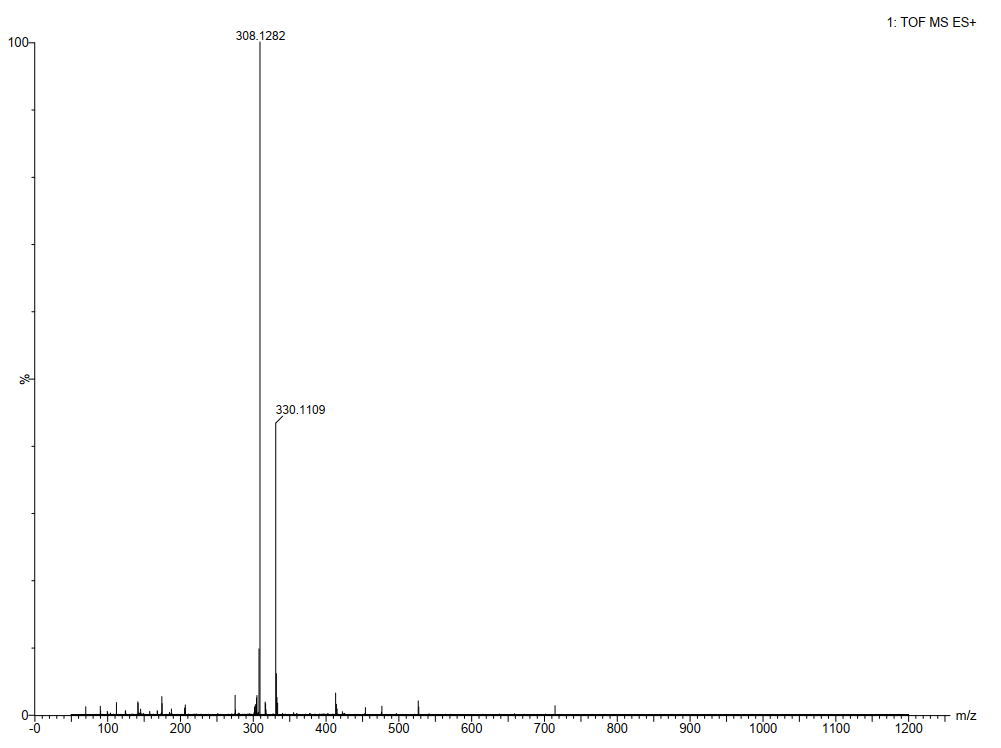


**Figure S14.**


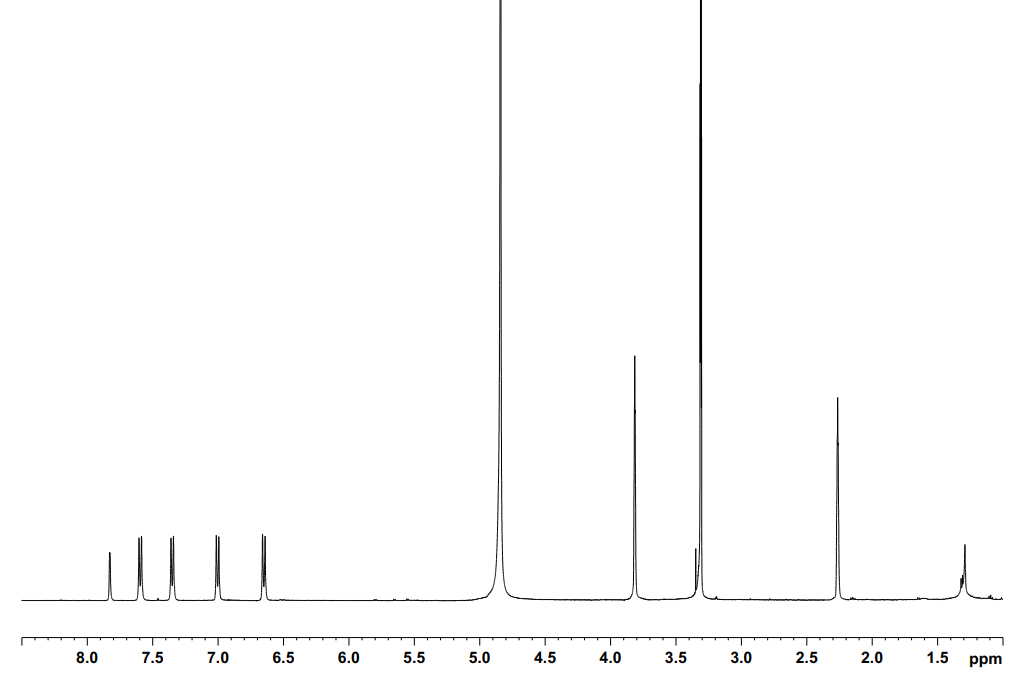


**Figure S15.**


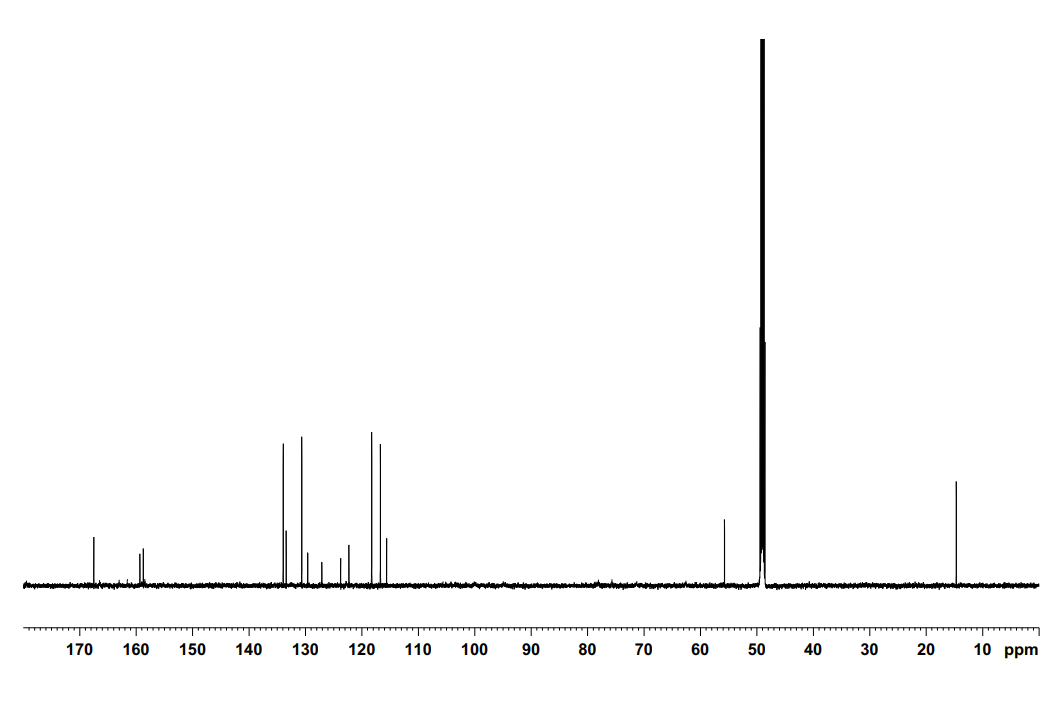


**Figure S16.**


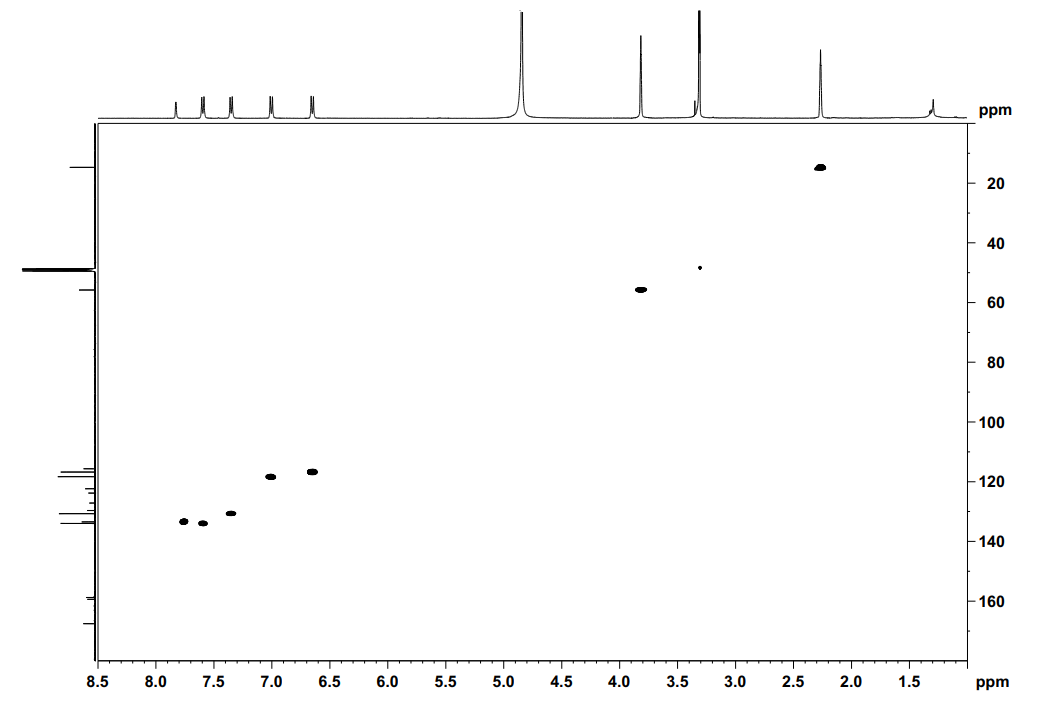


**Figure S17.**


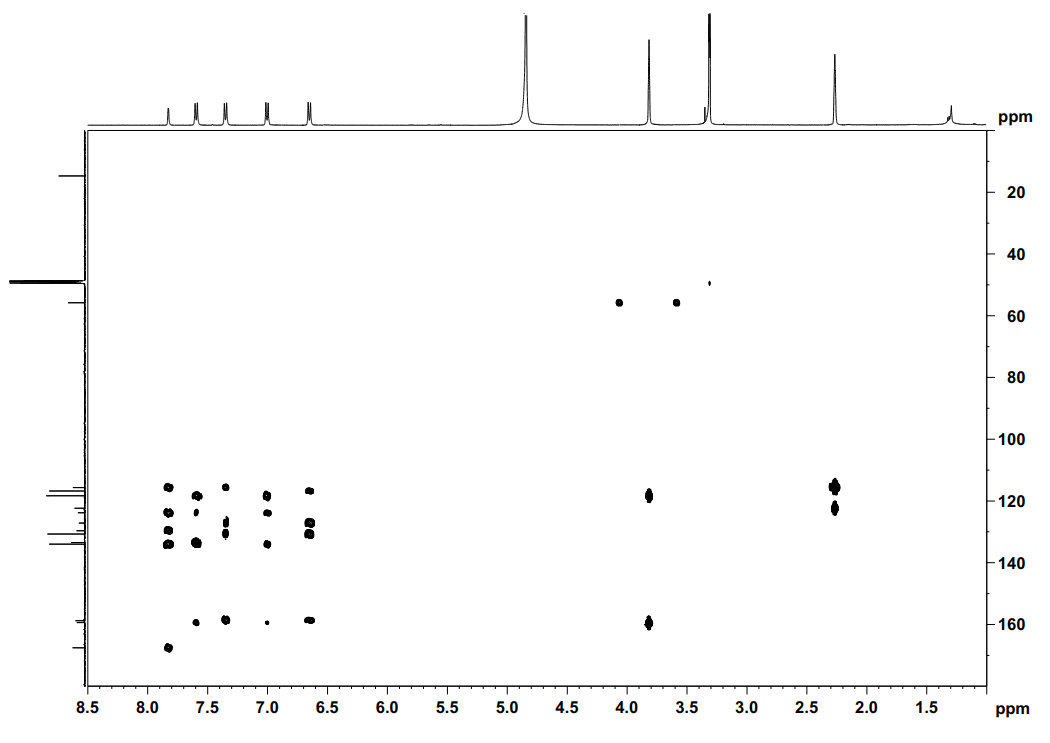


**Figure S18.**


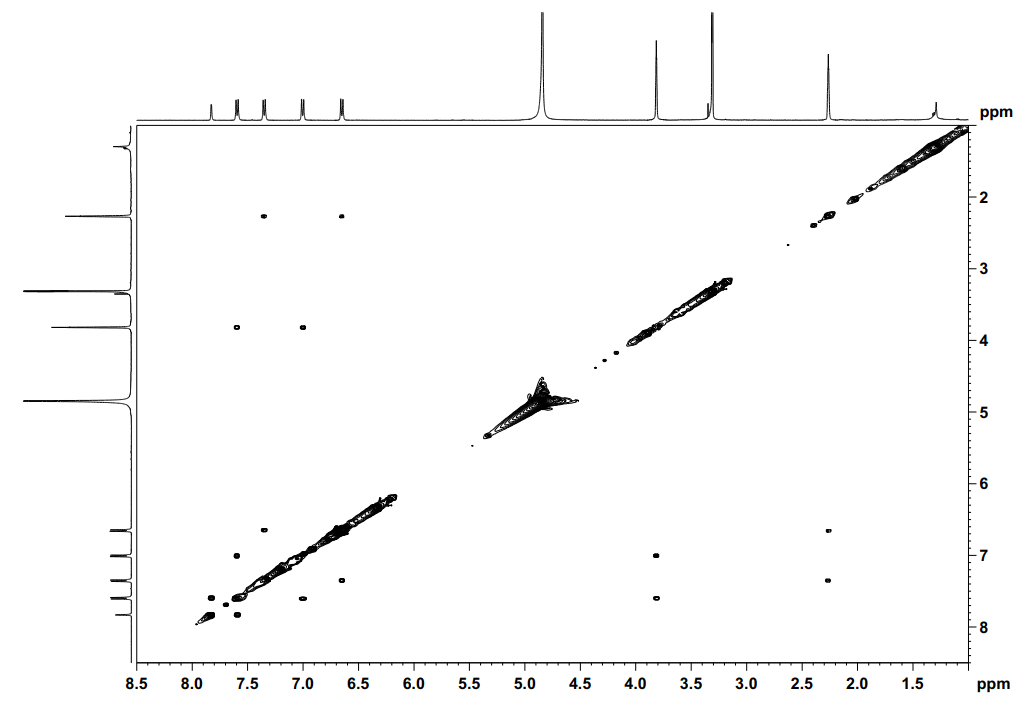


**Figure S19.**


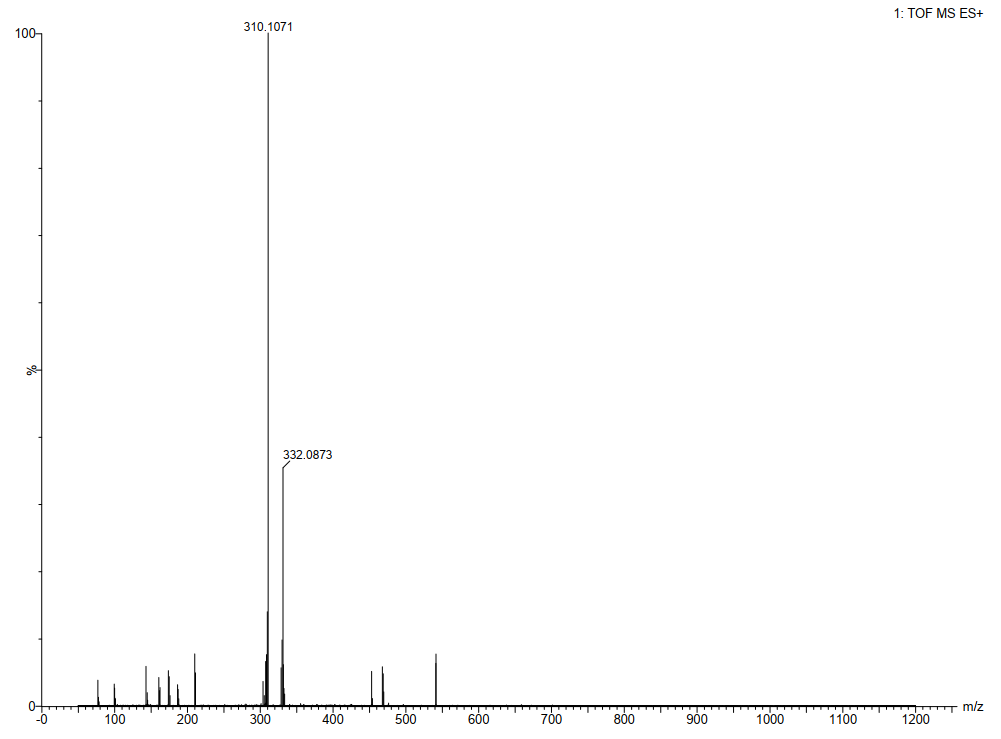


**Figure S20.**


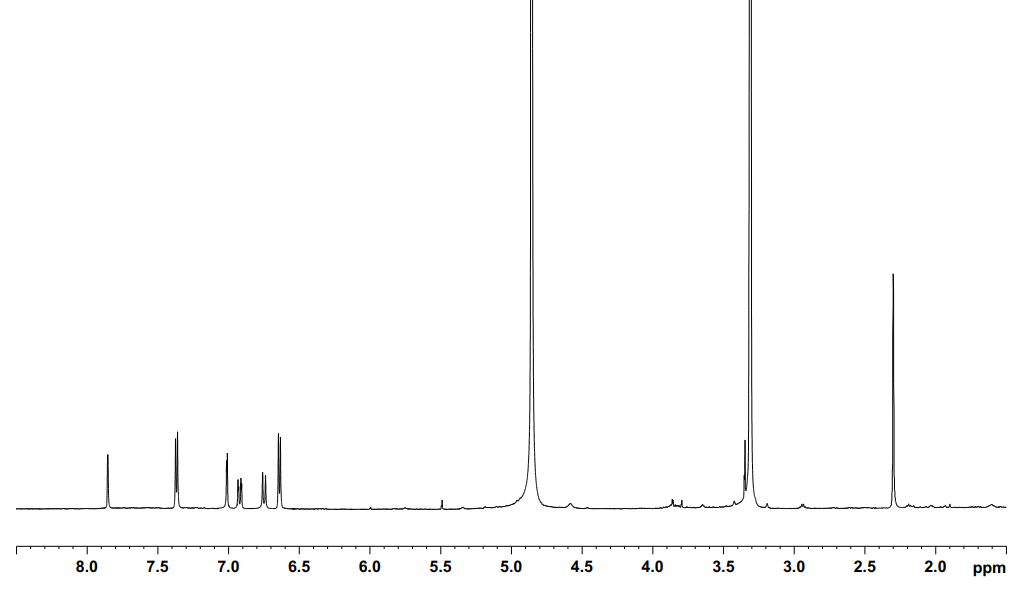


**Figure S21.**


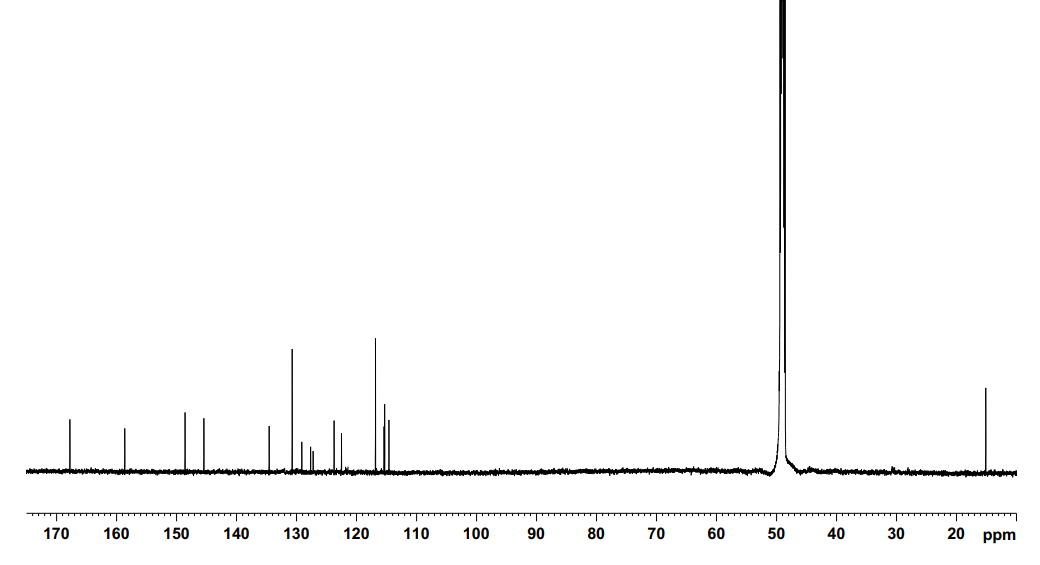


**Figure S22.**


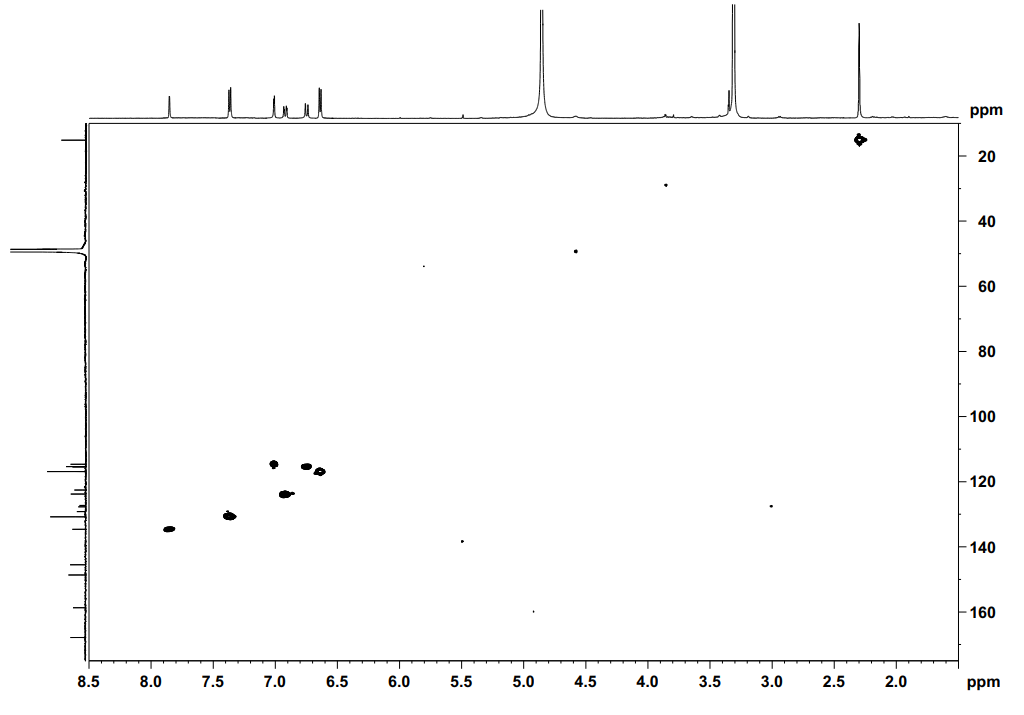


**Figure S23.**

.
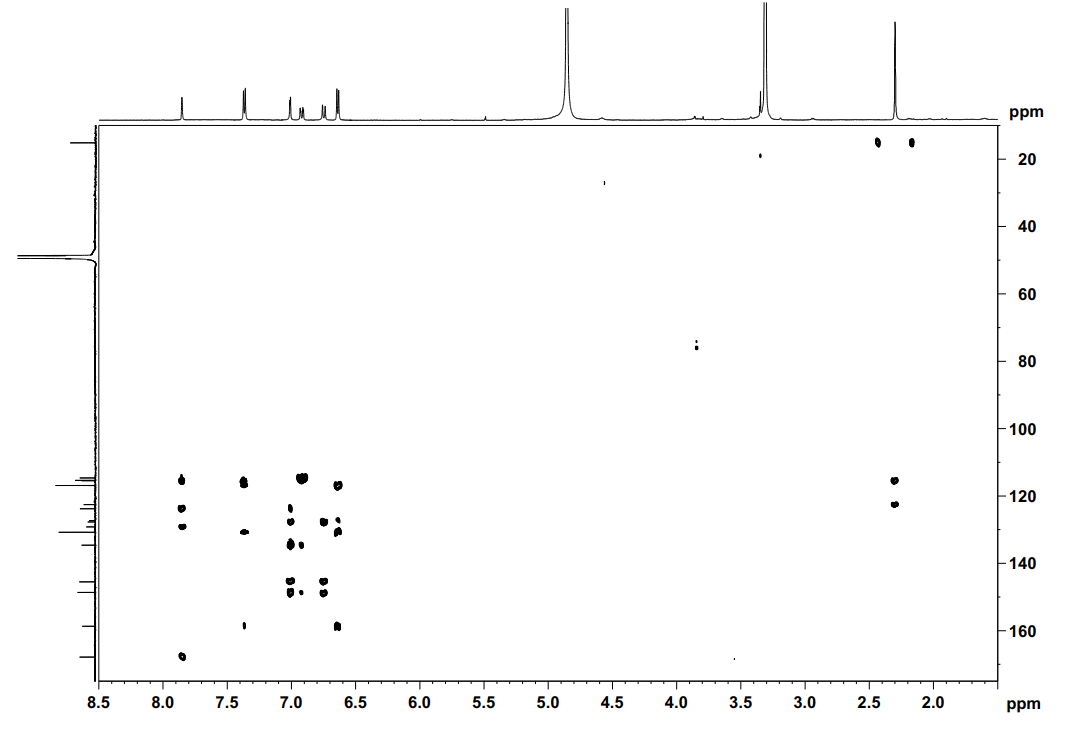


**Figure S24.**


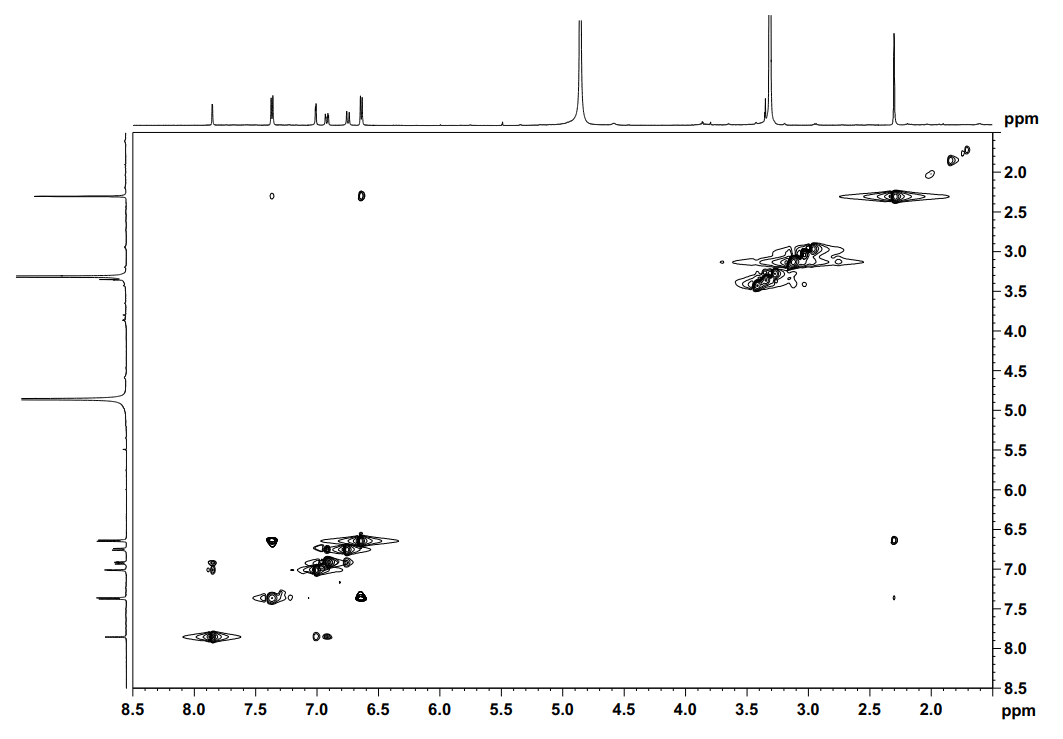


**Figure S25.**


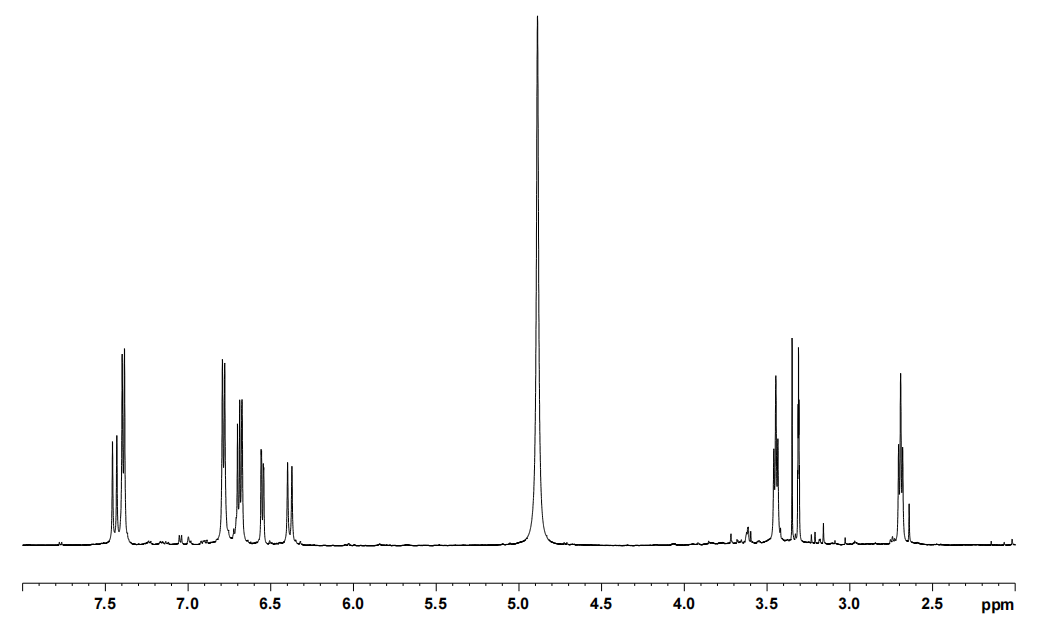


**Figure S26.**


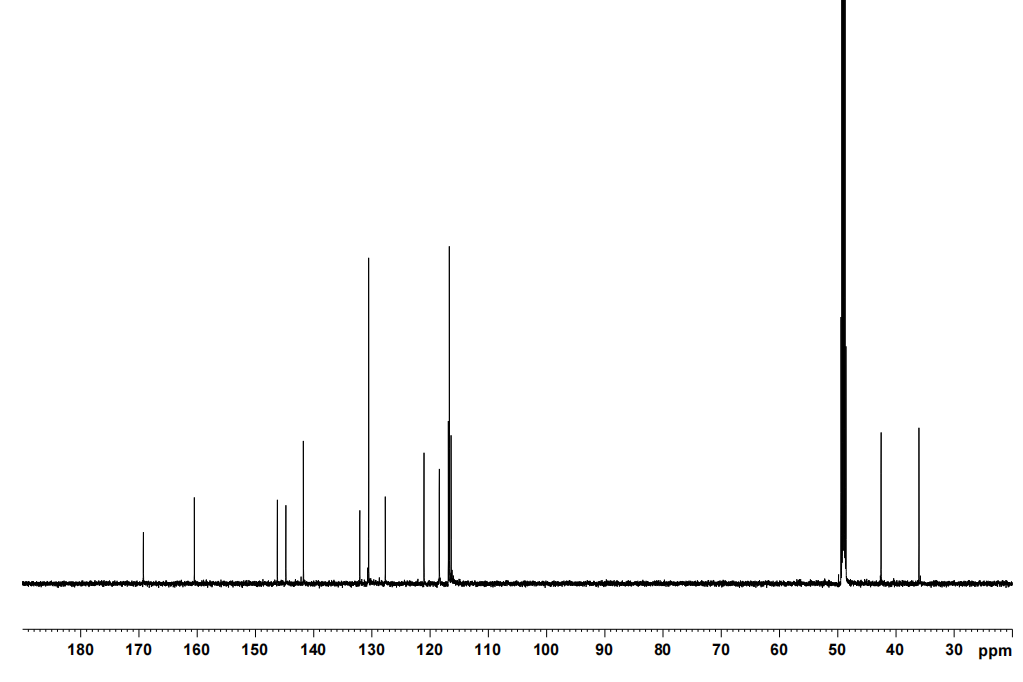


**Figure S27.**


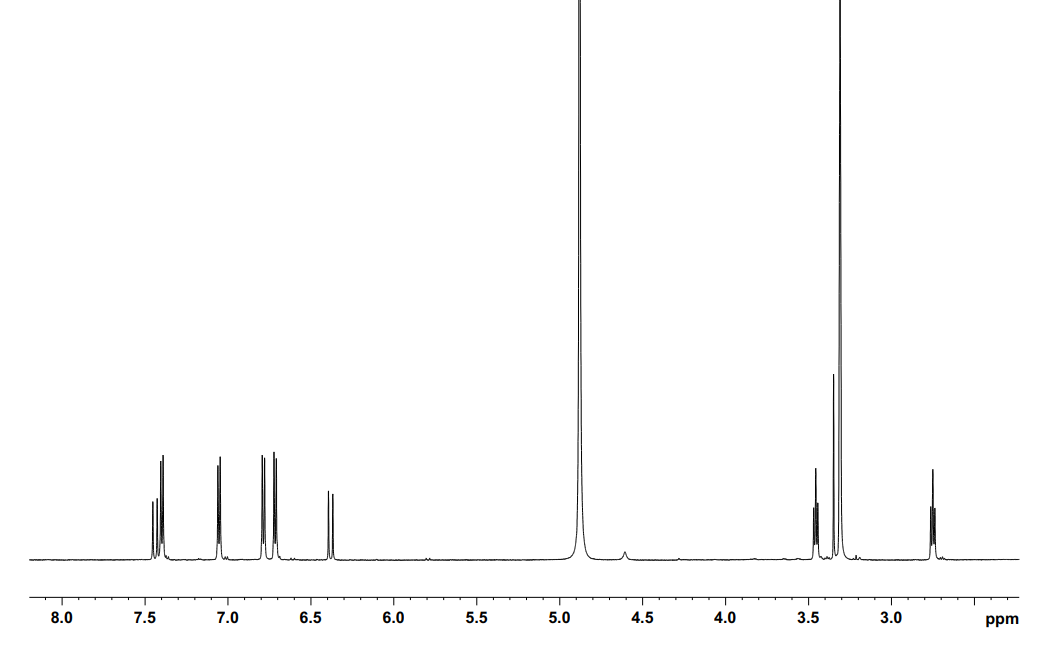


**Figure S28.**


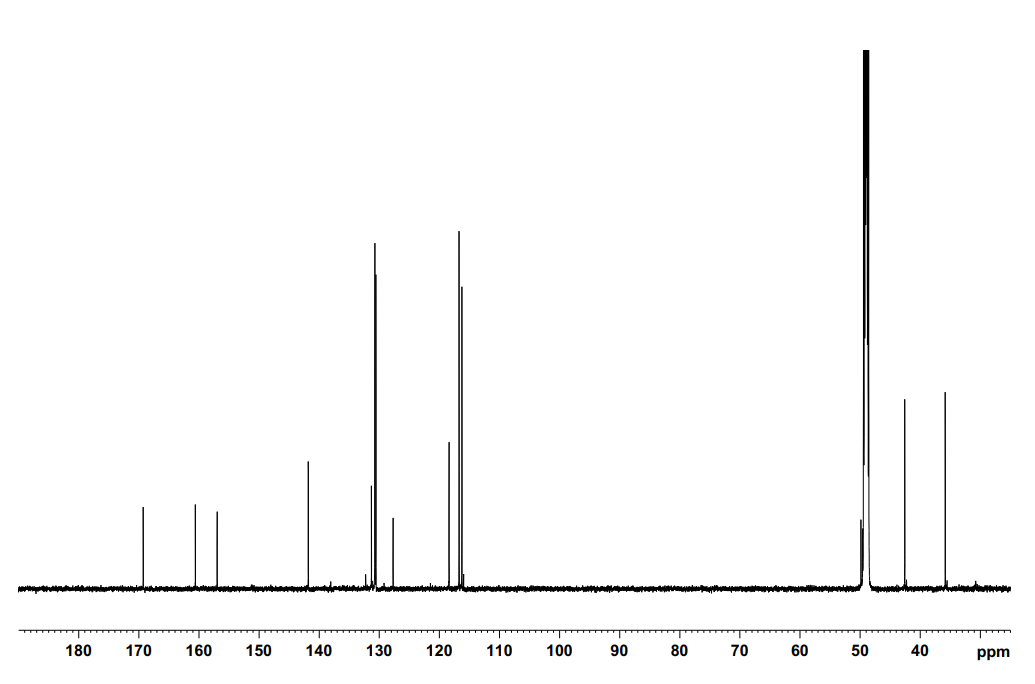


**Figure S29.**

**Table S1**. Moldock scores of the compounds with POD

| Ligand | Moldock Score (kcal/mol) |
| --- | --- |
|  | Protein (POD) |
| **1** | −121.391 |
| **2** | −128.591 |
| **3** | −125.039 |
| **4** | −135.462 |
| **5** | −136.321 |
| **6** | −131.036 |
